# Phosphorylation of the chromatin remodeling factor DPF3a induces cardiac hypertrophy through releasing HEY repressors from DNA

**DOI:** 10.1093/nar/gkv1244

**Published:** 2015-11-17

**Authors:** Huanhuan Cui, Jenny Schlesinger, Sophia Schoenhals, Martje Tönjes, Ilona Dunkel, David Meierhofer, Elena Cano, Kerstin Schulz, Michael F. Berger, Timm Haack, Salim Abdelilah-Seyfried, Martha L. Bulyk, Sascha Sauer, Silke R. Sperling

**Affiliations:** 1 Department of Cardiovascular Genetics, Experimental and Clinical Research Center, Charité – Universitätsmedizin Berlin and Max Delbrück Center for Molecular Medicine, 13125 Berlin, Germany; 2 Department of Biology, Chemistry and Pharmacy, Freie Universität Berlin, 14195 Berlin, Germany; 3 Group of Cardiovascular Genetics, Department of Vertebrate Genomics, Max Planck Institute for Molecular Genetics, 14195 Berlin, Germany; 4 DZHK (German Center for Cardiovascular Research), partner site Berlin, Berlin, Germany; 5 Max Planck Institute for Molecular Genetics, 14195 Berlin, Germany; 6 Division of Genetics, Department of Medicine, Brigham and Women's Hospital and Harvard Medical School, Boston, MA 02115, USA; 7 Committee on Higher Degrees in Biophysics, Harvard University, Cambridge, MA 02138, USA; 8 Hannover Medical School, Institute of Molecular Biology, Carl-Neuberg Str. 1, D-30625 Hannover, Germany; 9 Potsdam University, Institute of Biochemistry and Biology, Department of Animal Physiology, Karl-Liebknecht Str. 24-25, 14476 Potsdam-Golm, Germany; 10 Department of Pathology, Brigham and Women's Hospital and Harvard Medical School, Boston, MA 02115, USA; 11 CU Systems Medicine, University of Würzburg, 97080 Würzburg, Germany

## Abstract

DPF3 (BAF45c) is a member of the BAF chromatin remodeling complex. Two isoforms have been described, namely DPF3a and DPF3b. The latter binds to acetylated and methylated lysine residues of histones. Here, we elaborate on the role of DPF3a and describe a novel pathway of cardiac gene transcription leading to pathological cardiac hypertrophy. Upon hypertrophic stimuli, casein kinase 2 phosphorylates DPF3a at serine 348. This initiates the interaction of DPF3a with the transcriptional repressors HEY, followed by the release of HEY from the DNA. Moreover, BRG1 is bound by DPF3a, and is thus recruited to HEY genomic targets upon interaction of the two components. Consequently, the transcription of downstream targets such as *NPPA* and *GATA4* is initiated and pathological cardiac hypertrophy is established. In human, DPF3a is significantly up-regulated in hypertrophic hearts of patients with hypertrophic cardiomyopathy or aortic stenosis. Taken together, we show that activation of DPF3a upon hypertrophic stimuli switches cardiac fetal gene expression from being silenced by HEY to being activated by BRG1. Thus, we present a novel pathway for pathological cardiac hypertrophy, whose inhibition is a long-term therapeutic goal for the treatment of the course of heart failure.

## INTRODUCTION

Cardiac hypertrophy is defined as the increase in myocardial mass in response to pressure or volume stress, or mutations of sarcomeric proteins (1). Pathological hypertrophy represents a key risk factor for heart failure and accompanies nearly all forms of cardiovascular disease, including hypertension, hypertrophic cardiomyopathy (HCM) and aortic stenosis (AS). Sustained hypertrophy, which can eventually lead to heart failure, is associated with increased interstitial fibrosis, cell death and contractile dysfunction (2–4). Therefore, the prevention of pathological hypertrophy is of great therapeutic interest for cardiovascular disease.

On the cellular level, cardiac hypertrophy is characterized by an increase in cardiomyocyte size, with enhanced protein synthesis and changes to the sarcomere organization (5). Pathological stress is mediated via several intracellular signaling pathways that eventually activate the fetal gene program. The casein kinase 2 (CK2)-mediated signaling cascades have been shown to be important for the development of cardiac hypertrophy. CK2 is a typical serine/threonine kinase consisting of two catalytic subunits (αα, αα’ or α’α’) as well as two regulatory β subunits and has been implicated in many cellular and developmental processes (6). CK2α’, one of the catalytic subunits, is activated by the cardiac growth factor angiotensin II, which results in an imbalanced feedback loop between p27 and CK2α’ that is crucial for agonist- and stress-induced cardiac hypertrophic growth (7). Knockout of the other catalytic subunit CK2α in mice leads to structural defects in the heart and somites, with mice dying in mid-gestation (8). In response to hypertrophic stimuli, CK2α translocates to the nucleus and activates histone deacetylase 2 (HDAC2) by phosphorylating HDAC2 at serine 394 (9).

Cardiac hypertrophy is accompanied by up-regulation of the fetal gene program. Fetal genes are a set of genes that are often expressed only in the developing heart and are re-expressed during cardiac hypertrophy, and include natriuretic peptides (*NPPA, NPPB*), structure proteins (*β-MHC, α-skeletal actin*) and others (5). Transcriptional reprogramming of gene expression and reactivation of fetal genes are tightly controlled by transcription factors as well as chromatin modifying and remodeling factors (10–12).

The chromatin remodeling complexes alter chromatin structure using the free energy of ATP hydrolysis to change nucleosome positions relative to the DNA. The mammalian SWI/SNF-like complex (BAF complex) consists of 9–12 components, including the core component encoded by either brahma (BRM) or brahma-related gene 1 (BRG1) (13). Depletion of myocardial BRG1 causes a thin, compact myocardium and absent interventricular septum, and leads to embryonic lethality. In embryonic cardiomyocytes, BRG1 forms a complex with poly (ADP-ribose) polymerases (PARPs) to activate the fetal gene beta-myosin heavy chain (*β-MHC*), while it represses the cardiac contractile protein alpha-myosin heavy chain (*α-MHC*) via interaction with HDACs. Normally, BRG1 is turned off in the adult heart, but when the heart is stressed by disease or pressure overload, BRG1 is reactivated and interacts with HDACs and PARPs to repress *α-MHC* and activate *β-MHC* expression (14). A recent study showed that *Mhrt*, a cardiac specific long non-coding RNA, protects the heart from pathological hypertrophy via binding to BRG1, and thereby inhibiting chromatin targeting and gene regulation (15). Another muscle-specific component of the BAF complex is BAF60c (encoded by *SMARCD3*), which is highly expressed in heart and skeletal muscle during development (16). In skeletal muscle differentiation, phosphorylation of BAF60c by the p38α kinase promotes MyoD-BAF60c binding into the BAF complex, and thereby remodels chromatin and activates transcription of myogenic genes (17,18).

In 2008, we identified DPF3 (also named BAF45c) as a novel epigenetic factor for heart and muscle development. *DPF3* is significantly up-regulated in patients with Tetralogy of Fallot (TOF), which is characterized by structural cardiac defects and right ventricular hypertrophy (19,20). Morpholino knockdown of *dpf3* in zebrafish leads to impaired skeletal and cardiac muscle development and severely reduced ventricular contractility, with disassembled muscular fibers caused by transcriptional deregulation of structural and regulatory proteins (20). DPF3 consists of two distinct isoforms, namely DPF3a and DPF3b. The latter contains two plant homeodomains (PHD) that can bind to methylated and acetylated lysine residues of histone H3 and H4 (20), enabling a regulatory switch between poised and activated chromatin stages (21).

Here, we aim to shed light on the function of DPF3a, which is characterized by a half PHD finger and a specific C-terminus of so far unknown function. We show that DPF3, in particular DPF3a, is significantly up-regulated in pathologic cardiac hypertrophy in patients with HCM as well as AS. In response to hypertrophic stimuli, the protein kinase CK2 phosphorylates DPF3a that consequently binds and releases the transcriptional repressors HEY and recruits BRG1 to respective targets. Consequently, phosphorylated DPF3a (pDPF3a) initiates transcription of fetal genes promoting cardiac hypertrophy.

## MATERIALS AND METHODS

### Human samples

All cardiac samples were obtained with the approval of the ethics committee of the Charité – Universitätsmedizin Berlin and written informed consent was obtained for all individuals. Excised cardiac tissue from the left ventricular outflow tract was used in the study. It was obtained from four unrelated patients with AS undergoing aortic valve replacement, and from eight unrelated patients with HCM and severe obstruction of the left ventricular outflow tract undergoing transaortic subvalvular myotomy–myectomy. Both patient groups were characterized by severe hypertrophy of the left ventricle. Tissue from normal human hearts was obtained from unmatched organ donors without cardiac disease. All samples were snap frozen in liquid nitrogen after excision and stored at −80°C. Paraffin embedded TOF and normal heart samples were obtained as previously described (22).

### Human-induced pluripotent stem cell-derived cardiomyocytes (hiPSC-CMs)

The human-induced pluripotent stem cell line derived from a healthy individual was generously provided by the Stem Cell Core Facility of the Berlin Institute of Health (BIH). Cardiomyocyte differentiation was mediated by modulating Wnt-signaling, applying small molecules under fully defined conditions (23,24). Briefly, iPSCs were maintained in Essential 8 Media (Gibco) on Geltrex (Gibco) 6-well plates. iPSCs were dissociated using an EDTA-based passaging procedure (25) and 0.2–0.4 million cells were seeded in one 12-well plate containing Essential 8 media supplemented with 5 μM Pro-survival Compound, DDD00033325 (Calbiochem). After 24 h, Essential 8 media was exchanged and cells were maintained for an additional 24 h.

At day 0, differentiation was induced using RPMI/B27 without insulin supplemented with 9 μM GSK-3 inhibitor XVI (Calbiochem). Exactly 24 h later, media was changed to RPMI/B27 without insulin, and cells were subsequently cultivated for 48 h. On day 3, the differentiation media was exchanged for RPMI/B27 without insulin supplemented with 5 μM IWP2 (Calbiochem), and cells were cultivated for an additional 48 h. The media was then changed back to RPMI/B27 without insulin on day 5 of differentiation. From day 7 onwards, cells were cultivated in RPMI/B27 and media was exchanged every 3 days.

### Flow cytometry

After 15 days of differentiation, cells were washed twice with pre-warmed PBS and were singularized by applying 0.25% Trypsin/EDTA. Cells were fixed with 1% formaldehyde for 20 min at room temperature and were permealized using 90% ice-cold methanol and were subsequently stored at −20°C. For evaluation of differentiation efficiency, 10^5^ cells were evaluated. Briefly, cells were washed twice in PBS with 0.5% BSA (Flow Buffer 1) and incubated with the primary antibody against cardiac Troponin T (mouse IgG1, Lab Vision; 1:500) in PBS with 0.5% BSA and 0.1% Triton-X (Flow Buffer 2) at 4°C overnight. Afterwards, cells were washed with Flow Buffer 1 and were incubated with the secondary antibody Goat anti-Mouse IgG1 conjugated to Alexa Fluor 488 (1:1000) for 30 min at room temperature. Samples were washed twice with Flow Buffer 1 and were filtered through a 50 μm filter and evaluated on a BD FACS Canto II.

### Metabolic selection of hiPSC-CMs

As the analyzed population showed only an insufficient purity of 78% iPS-CMs metabolic purification applying lactate was performed as previously described (26). Briefly, cells were reseeded in lower density in RPMI supplemented with insulin, 20% Fetal Bovine Serum (FBS) and 5 μM Pro-survival Compound, DDD00033325 (Calbiochem) by applying 0.25% Trypsin/EDTA. Cells were allowed to recover for 4 days and were then cultivated in RPMI without Glucose and HEPES supplemented with 4 mM Sodium DL-lactate solution (Sigma) for 4 days. Afterwards, media was changed back to RPMI with insulin. All subsequent analyses were performed at a cell density of 37 500 cells/cm^2^.

### Cell culture

C2C12 myoblasts, H9C2 cardiomyocytes and HEK293T cells were cultured at 5% CO_2_ and 37°C in Dulbecco's Modified Eagle's Medium (Gibco) supplemented with 1% penicillin/streptomycin (Gibco) and 10% FBS (Biochrom). HL-1 cells were cultured in Claycomb medium (Sigma) containing 10% FBS, 100 mM norepinephrine, 4 mM L-glutamine and 1% penicillin/streptomycin.

For phenylephrine (PE) or Edothelin 1 (ET-1) treatment, HL-1, H9C2 or hiPSC-CMs were incubated with 10 μM PE (Sigma) or 1 nM ET-1 for 24 h. Cells were then harvested for RNA isolation or fixed with methanol for immunofluorescence staining.

### Isolation and transfection of mouse primary cardiomyocytes

Neonatal hearts were isolated from 1–3 days old mice. After removing atria and large arteries, ventricles were minced and treated with collagenase buffer for 1 h at room temperature with gently shaking. The enzyme digestion was stopped by the addition of 10% FBS containing DMEM. Fibroblasts were removed twice by 1 h pre-plating. The cardiomyocytes were counted and plated on collagen-coated culture dishes and were maintained in 10% FBS in DMEM without antibiotics. Isolated mouse primary cardiomyocytes were cultured for 3 days and then transfected with Flag-tagged DPF3a WT and S348A mutant constructs using Lipofectmine 3000 (Invitrogen).

### Recombinant protein expression and purification

GST fusion proteins were expressed in *E. coli* BL21 DE3 pRARE3 and purified using Glutathione–Sepharose matrix (GE Healthcare) according to the manufacturer's instructions.

### GST pull-down

GST-CK2α/α’/β fusion proteins were coupled to Glutathione–Sephharose matrix, according to the manufacturer's indication (GE Healthcare), and subsequently incubated with Flag-DPF3a/b recombinant proteins for 2 h at 4°C in binding buffer (4.2 mM Na_2_HPO_4_, 2 mM KH_2_PO_4_, 250 mM NaCl, 10 mM KCl, 0.1% NP40, 0.5% BSA, pH 7.2, complete protease inhibitor (Roche)). Matrices were washed three times in binding buffer, resuspended in 4x LDS sample buffer, denatured at 95°C for 5 min and subjected to Western blot analysis.

### Phosphorylation prediction and conservation analysis of DPF3a

The multiple alignment of DPF3a in different species was generated using ClustalW2 (27). Phosphorylation site prediction was performed using NetPhos 2.0 Server (28) with standard parameters. CK2-specific phosphorylation site prediction was carried out using KinasePhos 2.0 (29) with standard parameters.

### Mass spectrometry analysis

Tandem affinity purification followed by mass spectrometry was carried out as described previously (20). To study the phosphorylation of DPF3a, HEK293T cells were transfected with Flag-DPF3a. Cell extracts were immunoprecipitated with anti-Flag M2 antibody (Sigma) and immunoprecipitated complexes were digested in solution. Phosphopeptide enrichment was conducted using the Titansphere Phos-TiO kit (GL Sciences). Mass spectrometry analysis for identification of proteins and for detection of protein phosphorylation was performed as previously described (30,31).

### *In vitro* kinase assay

*In vitro* phosphorylation of recombinant proteins was performed as described previously (32). Briefly, 1 μg of GST fusion proteins were incubated with recombinant active CK2 (NEB) and ATPγS (Epitomics) in the reaction buffer at 30°C for 2 h. Proteins were then alkylated with 2.5 mM p-Nitrobenzyl mesylate (PNBM, Epitomics) for 2 h at room temperature and the products were analyzed by Western blot using an anti-Thiophosphate ester antibody (Epitomics). The CK2-specific inhibitor tetrabomocinnamic acid (TBCA, Millipore) was used to abolish CK2 activity in the *in vitro* kinase assay.

### Generation of anti-pDPF3a antibody

The DPF3a antibody that can specifically recognize phosphorylated S348 (anti-pDPF3a) was generated in rabbit by Thermo Fisher Scientific. The phosphorylated peptide against the epitope CRRSGRG[pS]PTADK of DPF3a was used.

### Co-immunoprecipitation (CoIP)

HEK293T cells were transiently transfected with the indicated expression vectors for Flag- or HA-tagged proteins using polyethylenimine (PEI). The cells were lysed in CoIP buffer (20 mM Tris-HCl pH 7.4, 150 nM NaCl, 1 mM EDTA, 1% Triton, 1 mM DTT, 0.1 mM PMSF, 1 mM NaVO4, protease inhibitor (Roche) and phosphatase inhibitor (Roche)). Protein concentrations were determined using the Bradford assay (Sigma). A total of 500 μg cell extracts were incubated with Flag M2 matrix (Sigma) or HA-matrix (Roche) for 2 h at 4°C. The matrix was then washed three times with ice-cold CoIP buffer and eluted for Western blot.

### Western blotting and quantification

Cells and human cardiac biopsies were treated with lysis buffer (20 mM Tris-HCl pH 7.4, 150 nM NaCl, 1 mM EDTA, 1% Triton, 1 mM DTT, 0.1 mM PMSF, protease inhibitor (Roche), 1 mM NaVO_4_) for protein extraction. Western blot was performed according to standard protocols. All antibodies, with their respective dilutions, are given in Supplementary Table S1. Image Lab 5.0 (Bio-Rad) was used to quantify the blot signals.

### Immunofluorescence staining

For cell immunofluorescence analysis, the cells were fixed with methanol. Blocking was carried out in 3% normal goat serum in PBS for 1 h at room temperature. Primary and secondary antibodies were applied in the same buffer for 2 h at room temperature, each followed by three washes in PBS and a DAPI counterstaining. The cells were mounted in Mowiol 4–88 (Carl Roth) and examined on a LSM 710 confocal microscope (Carl Zeiss). The cell size of transfected cells was measured using ImageJ.

For human tissue immunofluorescence analysis, paraffin-embedded right ventricular biopsies of healthy and TOF individuals were rehydrated and subjected to heat-mediated antigen retrieval and quenching of endogenous peroxidase activity. Blocking was performed with donkey serum (10%) and BSA (2%) in PBT (0.3%) and primary antibodies were applied overnight at 4°C in the same buffer. Corresponding secondary antibodies were subsequently applied, and the tissue incubated for 1 h at room temperature; this was followed by application of Vectastain Elite ABC Kit (Vector Lab) and TSA Plus Cyanine-5 System (Perkin Elmer) according to the manufacturer's instructions. Nuclei were counterstained with DAPI and slides mounted with Fluoromount (Sigma). Omission of the first antibody served as a negative control. All images were captured using a Leica SP5 confocal microscope. Antibodies and reagents with their respective dilutions are listed in Supplementary Table S1.

### RNAi knockdown

For RNAi knockdown, C2C12 cells or hiPSC-CMs were transfected with siRNAs (Supplementary Table S2) targeting *Dpf3a, Hey1* and *Ck2α*, respectively. As a control, the cells were transfected with an unspecific siRNA (siNon). Cells were grown to 50% confluence for at least 2 days without addition of antibiotics. A total of 1 × 10^5^ cells were seeded into 6-well plates with 2 ml media resulting in 40% confluence after 4 h. The mixture of 4.4 μl (20 μM) siRNA in 100 μl of DMEM media and 8.8 μl Lipofectamine 3000 (Invitrogen) in 100 μl DMEM media was incubated for 15 min at room temperature and added drop-wise to the cells. The cell culture medium was changed after 24 h and cells were harvested for protein extraction or RNA preparation after 48 h.

### Gene expression analysis

Total RNA of cultured cells and human cardiac biopsies was isolated using TRIzol reagent (Invitrogen) followed by DNase digest (Promega) and ethanol precipitation according to standard protocols. Reverse transcription reactions were carried out via AMV-RT (Promega) with random hexamers (Amersham Pharmacia Biotech). Quantitative real-time PCR measurements were performed using the GoTaq qPCR Master Mix (Promega) and the ABI PRISM 7900HT Sequence Detection System. Gene expression was calculated using the ΔCT method with normalization to the housekeeping gene *HPRT* or *GAPDH*. Primer sequences are given in Supplementary Table S3.

### Chromatin immunoprecipitation (ChIP) and qPCR

ChIP experiments with C2C12 cells were carried out with the MAGnify™ Chromatin Immunoprecipitation System (Life Technologies) following the manufacturer's instructions with some modifications. Sonication was performed using the Bioruptor UCD300 (Diagenode) to obtain chromatin fragments of approximately 100–500 bp. For Hey1 ChIP, C2C12 cells were transiently transfected with Flag-Hey1 and empty Flag vector, which was used as a control. Anti-pDPF3a and anti-Flag M2 antibodies were used in ChIP experiments. ChIP DNA was purified using the Zymo ChIP DNA cleanup kit. ChIP results were analyzed by quantitative PCR. ChIP enrichment was normalized to the input and expressed as relative enrichment of the material precipitated by the indicated antibody on target regions. ChIP primers are listed in Supplementary Table S4.

### Site-directed mutagenesis

Site-directed mutagenesis of DNA was performed using the QuikChange II site-directed mutagenesis kit (Agilent) according to manufacturer's instructions. Oligonucleotides for mutagenesis were designed to introduce mutations of the DPF3a phosphorylation sites. Mutagenesis was confirmed by plasmid sequencing (Eurofins Genomics).

### Reporter gene assay

For luciferase assays, approximately 10^4^ HEK293 cells were transiently transfected with 50 ng of reporter vector, 5 ng of Renilla luciferase vector for internal normalization of transfection efficiency and 50–150 ng of the respective expression vectors. Activity was measured by Dual-Luciferase assay (Promega) after 48 h in a Centro LB960 Luminometer (Berthold, Bad Wildbad, Germany). All measurements were performed in triplicates.

## RESULTS

### *DPF3* is significantly up-regulated in hypertrophic hearts

**Figure 1.**
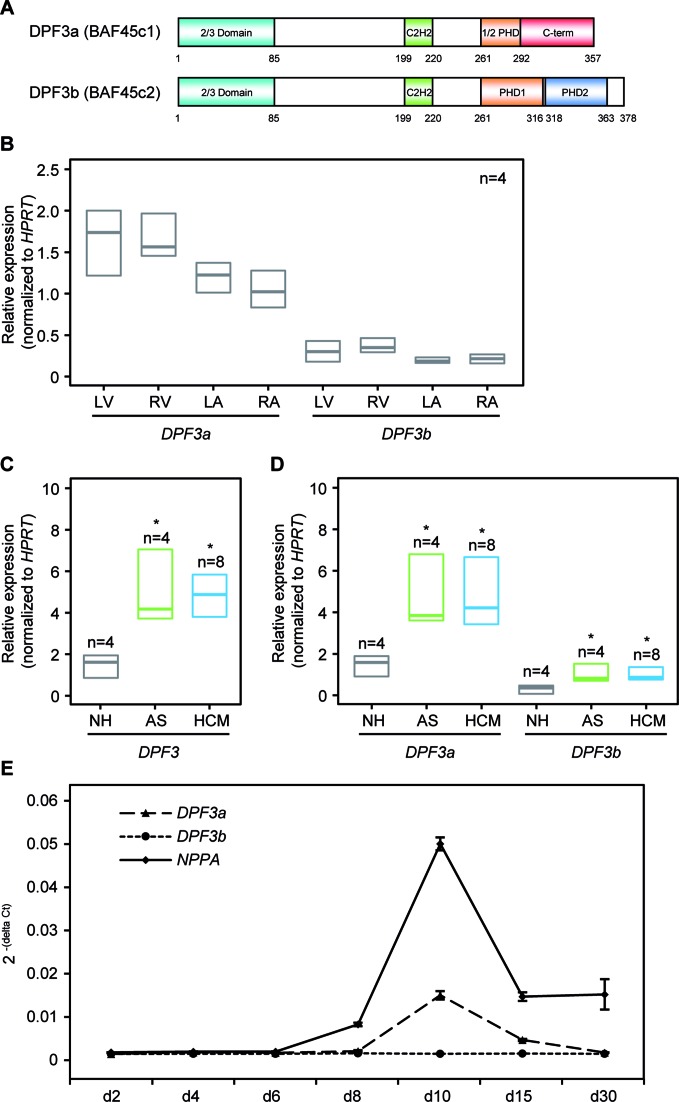
Up-regulation of *DPF3* in hypertrophic hearts. (**A**) Schematic representation of the DPF3 isoforms. The isoform DPF3a contains a half PHD finger and a specific C-terminus, whereas DPF3b contains a double PHD finger. Amino acid positions are depicted. Teal: 2/3 domain; green: C2H2-Krüppel-like zinc finger; orange: first plant homeodomain; blue: second plant homeodomain; red: DPF3a specific C-terminus. (**B**) Expression analysis of *DPF3a* and *DPF3b* splice variants in normal human heart tissues (n = 4) of left ventricle (LV), right ventricle (RV), left atrium (LA) and right atrium (RA) using qPCR. Results represent median expression levels with 25% and 75% quartile; assays were performed in triplicates. (**C**) Expression of *DPF3* mRNA in hypertrophic and healthy hearts. qPCR analysis of *DPF3* mRNA levels in myocardial, left ventricular tissue from patients with aortic stenosis (AS), hypertrophic cardiomyopathy (HCM) and normal hearts (NH). (**D**) Analysis of splice variants expression of *DPF3a* and *DPF3b* in AS, HCM and NH. Statistically significant differences were analyzed using a two-sided Student *t*-test (*P < 0.05). (**E**) Temporal expression profiles of *DPF3a, DPF3b* and *NPPA* during differentiation of human-induced pluripotent stem cell-derived cardiomyocytes (hiPSC-CMs). qPCR was performed on samples obtained from different days during cardiac differentiation. Expression values were normalized to the housekeeping gene *GAPDH*.

In mammals, *DPF3* occurs in two splice variants, namely *DPF3a* and *DPF3b*. DPF3a (BAF45c1) is characterized by a functionally unknown half PHD finger followed by a unique C-terminus, whereas DPF3b (BAF45c2) comprises a C-terminus harboring a double PHD finger previously characterized by us and others (Figure 1A) (20,21). Both variants are expressed in all four chambers of the normal human adult hearts with *DPF3a* being continuously higher expressed than *DPF3b* (Figure 1B).

Previously, we showed that both *DPF3a* and *DPF3b* are significantly up-regulated in the hypertrophic right ventricle of TOF hearts (19,20). To further investigate the role of *DPF3* in pathological cardiac hypertrophy, we studied cases of HCM and pressure overload related hypertrophy resulting from AS. The details of all human heart samples are given in Supplementary Table S5. Quantitative real-time PCR showed a significant up-regulation of *DPF3* as well as the two individual splice variants in the left ventricle of HCM and AS cases compared to normal hearts (NH) (*P* < 0.05; Figure 1, C and D). Pathological cardiac hypertrophy is regulated by re-activation of fetal gene expression. To test if *DPF3* is a fetal-like gene, we analyzed the expression of *DPF3a* and *DPF3b* during differentiation of human-induced pluripotent stem cell-derived cardiomyocytes (hiPSC-CMs). The differentiation process and purity of hiPSC-CMs was characterized by marker gene analysis and flow cytometry (Supplementary Figure S1). Interestingly, *DPF3a* shows a similar expression profile to the fetal gene *NPPA* with the highest expression at day 10, whereas *DPF3b* is expressed at a very low level during the differentiation process (Figure 1E). Taken together, all these results suggest that *DPF3a* can be considered as a fetal-like gene, which might play a role in pathological cardiac hypertrophy in general.

### CK2 binds and *in vitro* phosphorylates DPF3a

To elucidate the role of DPF3 in cardiac hypertrophy, we first searched for DPF3 interacting proteins in HEK293 cells. Using the tandem affinity purification (TAP) technique followed by mass spectrometry (MS) analysis, we identified 11 and 6 proteins specifically interacting with DPF3a or DPF3b, respectively, with a Mascot score >40 and a minimum number of two matching spectra (Table 1). All subunits of the tetrameric casein kinase 2 complex, CK2α, CK2α’ and CK2β were found as exclusive interaction partners of DPF3a (Table 1). None of the CK2 subunits was identified when DPF3b was used as the bait protein.

**Table 1.** DPF3a and DPF3b specific interaction partners in cytoplasm

Bait protein	Hugo ID	Accession number	MW (Da)	Mascot score	Matching spectra	Protein name
DPF3a	CSNK2A1	P68400	45 229	459	13	Casein kinase II subunit alpha
	CSNK2A2	P19784	41 358	251	10	Casein kinase II subunit alpha'
	CSNK2B	P67870	25 268	54	5	Casein kinase II subunit beta
	ASCC3L1	O75643	246 006	56	3	activating signal cointegrator 1 complex subunit 3-like 1
	UBR4	Q5T4S7	580 547	49	8	Zinc finger UBR1-type protein 1, ubiquitin protein ligase E3 component n-recognin 4
	UBR5	O95071	312 352	49	5	E3 ubiquitin-protein ligase EDD1
	HDGFRP3	Q9Y3E1	22 663	101	3	Hepatoma-derived growth factor-related protein 3
	HSPA1L	P34931	70 730	71	6	Heat shock 70 kDa protein 1L
	AIFM1	O95831	67 144	41	4	Apoptosis-inducing factor 1, mitochondrial precursor
	IGKC	P01834	11 773	61	2	Ig kappa chain C region
	GPSN2	Q9NZ01	36 410	51	4	Synaptic glycoprotein SC2
DPF3b	TUBA8	Q9NY65	50 746	75	9	Tubulin alpha-8 chain
	XPOT	O43592	111 148	41	5	Exportin-T
	TUBA3E	Q6PEY2	50 626	444	13	Tubulin alpha-3E chain
	EEF1A2	Q05639	50 780	51	3	Elongation factor 1-alpha 2
	TIMM50	Q3ZCQ8	39 850	143	3	Import inner membrane translocase subunit TIM50, mitochondrial precursor
	AHSA1	O95433	38 421	48	2	Activator of 90 kDa heat shock protein ATPase homolog 1

Protein specifically associated with DPF3a or DPF3b in the cytoplasm identified by tandem affinity purification followed by mass spectrometry. Da: Dalton, MW: molecular weight.

**Figure 2.**
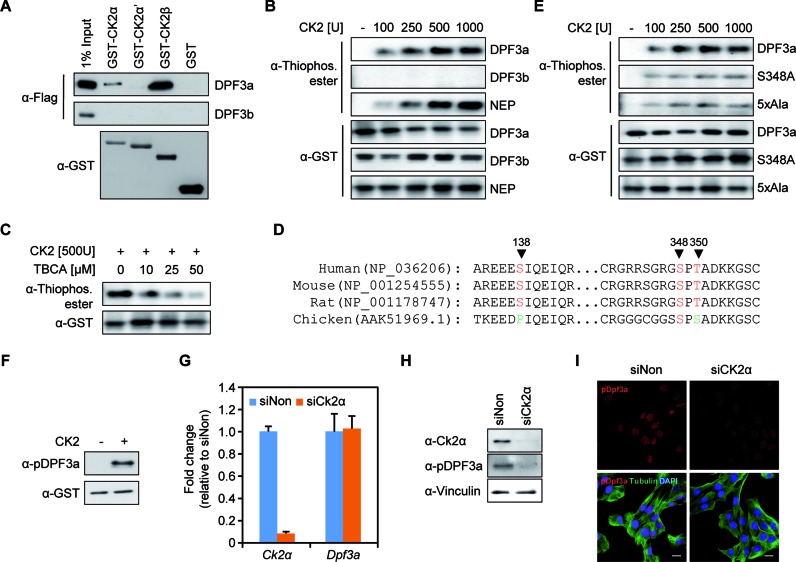
CK2 binds and phosphorylates DPF3a at S348. (**A**) GST pull-down with GST-CK2 subunits and Flag-DPF3a/b. Similar expression of GST levels could be shown for the CK2 subunits and the control GST vector. GST pull-down results show an interaction of the CK2α and β subunit with full-length DPF3a. (**B**) *In vitro* CK2 kinase assay with recombinant CK2 and purified GST fusion DPF3a. Increasing amount of CK2 was incubated with GST-DPF3a and combinations of ATPγS and p-nitrobenzyl mesylate (PNBM) as indicated; reaction products were analyzed by Western blot with anti-Thiophosphate ester antibody. GST-DPF3b and GST-NEP were used as the negative and positive control, respectively. Immunoblotting against GST demonstrates that a similar amount of protein was used for each condition. (**C**) The CK2 specific inhibitor, tetrabromocinnamic acid (TBCA), was used in the kinase assay and effectively reduced DPF3 phosphorylation. (**D**) Sequence conservation of DPF3a peptides surrounding the identified phosphorylation sites in human, mouse, rat and chicken. (**E**) GST-DPF3a WT, S348A mutant or 5xAla mutant were *in vitro* phosphorylated by CK2 before blotting. (**F**) GST-DPF3a was phosphorylated by CK2 and detected using the anti-pDPF3a antibody in immunoblotting. (**G**) Knockdown of Ck2α in C2C12 cells using siRNA. Expression of *Ck2α* and *Dpf3a* after Ck2α siRNA knockdown was analyzed by qPCR. (**H**) The protein level of Ck2α and pDPF3a was was analyzed by immunoblotting in Ck2α knockdown situation. (**I**) Immunofluorescence analysis of pDPF3a in Ck2α knockdown situation in C2C12 cells. C2C12 cells were transfected with Ck2α siRNA for 48 h. The cells were then fixed with methanol and stained with antibody against pDPF3a (red) and antibody against Tubulin (green). Scale bar, 20 μm.

This observation led us to investigate whether DPF3a could directly associate with CK2α/α’/β *in vitro*. Thus, we performed *in vitro* interaction studies with purified proteins, using GST-tagged purified CK2α/α’/β in combination with Flag-tagged DPF3a/b. Pull-down assays showed that DPF3a strongly interacts with CK2β and weakly binds to CK2α (Figure 2A). This is in line with the fact that CK2α is the catalytic subunit, whereas CK2β is the regulatory subunit of CK2 and responsible for substrate recognition (6). Again, we observed no interactions between CK2 subunits and DPF3b (Figure 2A). CK2 is a well-known ubiquitously expressed and evolutionarily conserved serine/threonine protein kinase (6). To investigate whether DPF3a is phosphorylated by CK2, we applied *in vitro* kinase assays using purified GST-fusion protein of DPF3a, with GST-DPF3b and GST-NEP serving as negative and positive control, respectively (33). Both DPF3a and NEP, but not DPF3b, were *in vitro* phosphorylated by CK2 and DPF3a phosphorylation became saturated when 500U CK2 was applied in the kinase assay (Figure 2B). Moreover, DPF3a phosphorylation is reduced upon treatment with increasing amounts of the CK2 specific inhibitor TBCA (Figure 2C), indicating that DPF3a phosphorylation *in vitro* is largely dependent on CK2 activity.

### S348 of DPF3a is phosphorylated by CK2 *in vivo*

In order to analyze which residues of DPF3a are phosphorylated by CK2, we used the prediction tools Netphos 2.0 (28) and KinasePhos 2.0 (29). We found potential phosphorylation sites for the full DPF3a sequence and revealed several sites in the DPF3a-specific C-terminus. Here, multiple minimal CK2 consensus motifs (S/T-X-X-E/D) were predicted between aa293 and aa357 (T309, T310, S316, S318 and S348) (Supplementary Figure S2). The DPF3a specific C-terminus is highly conserved across vertebrate and mammalian species (Supplementary Figure S3). To confirm potential phosphorylation sites, we analyzed the phosphorylation of DPF3a in HEK293 cells using immunoprecipitation followed by mass spectrometry analysis. In total, we identified three phosphorylation sites in DPF3a, namely S138, S348 and T350 (Table 2). Of these, in particular S348 is conserved in human, mouse and chicken (Figure 2D), which suggests a potential functional role of this site. Therefore, we studied the CK2 dependent phosphorylation of DPF3a S348 using an *in vitro* kinase assay on purified GST-fusion protein in which serine 348 was mutated to alanine (S348A). We found that phosphorylation of DPF3a by CK2 is significantly reduced by the mutation of S348A compared to the wild-type (WT) DPF3a (Figure 2E). We also mutated all five predicted CK2 mediated phosphorylation sites (5xAla) and observed a further reduction of DPF3a phosphorylation (Figure 2E), suggesting additional functional phosphorylation sites *in vitro*.

**Table 2.** Phosphorylated sites in DPF3a

Peptide	Mascot score	PEP	Position
_EEES(ph)IQEIQR_	171.99	2.08E-05	138
_GS(ph)PTADKK_	108.43	2.05E-06	348
_GSPT(ph)ADKK_	130.66	1.83E-11	350

Phosphorylated sites within DPF3a identified by mass spectrometric analysis. PEP: posterior error probabilities.

Furthermore, we generated a DPF3a antibody that can specifically recognize phosphorylated S348 (anti-pDPF3a). The specificity of the anti-pDPF3a antibody was confirmed by Western blot and immunofluorescence with peptide control assays (Supplementary Figure S4). In immunofluorescence staining, we found that pDpf3a is exclusively present in the nucleus in HL1 and C2C12 cells (Supplementary Figure S4, Figure 2I). Further, we could verify the *in vitro* phosphorylation of Dpf3a S348 by Ck2 using the anti-pDPF3a antibody (Figure 2F). In order to investigate phosphorylation of S348 *in vivo*, we knocked down Ck2 using RNA interference in C2C12 myoblasts. Treatment with siCk2α significantly reduced Ck2α mRNA and protein expression (Figure 2, G and H). Elimination of Ck2α function did not affect the expression of Dpf3a (Figure 2G). Notably, the phosphorylation of DPF3a at S348 was abolished in Ck2α knockdown (Figure 2, H and I), suggesting that S348 is *in vivo* phosphorylated by Ck2.

### Phosphorylation of S348 is essential for cardiac hypertrophy

**Figure 3.**
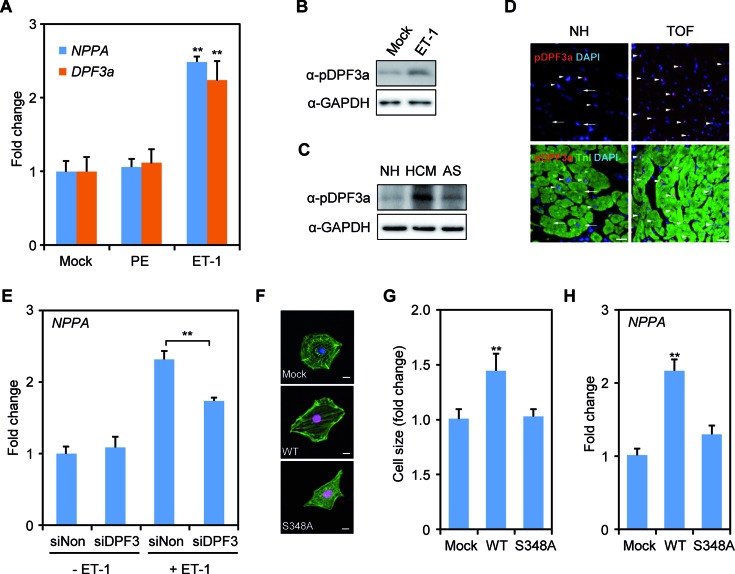
DPF3a S348 phosphorylation is important for cardiac hypertrophy. (**A**) *NPPA* and *DPF3a* mRNA expression was analyzed in human-induced pluripotent stem cell-derived cardiomyocytes (hiPSC-CMs) after phenylephrine (PE) or endothelin 1 (ET-1) treatment for 24 h. (**B**) Immunoblotting analysis of the pDPF3a protein level with anti-pDPF3a antibody in ET-1 induced hypertrophic hiPSC-CMs. (**C**) The protein level of pDPF3a was analyzed in the hearts of patients with hypertrophic cardiomyopathy (HCM) and aortic stenosis (AS). (**D**) Immunofluorescence of paraffin-embedded right ventricular biopsies of normal hearts (NH) and Tetralogy of Fallot (TOF) individuals. Red: pDPF3a; green: Troponin I (TnI); arrow: pDPF3a positive cardiomyocytes; arrow with tail: pDPF3a negative cardiomyocytes. Scale bar, 25 μm. (**E**) siRNA knockdown of DPF3 followed by ET-1 treatment in hiPCS-CMs. Expression of *NPPA* after DPF3 siRNA knockdown and ET-1 treatment was analyzed by qPCR. (**F**) Mouse primary cardiomyocytes were transfected with either Flag-tagged DPF3a WT or S348A constructs. Cells were then stained with antibody against Flag (red), and antibody against Actinin (green). Scale bar, 10 μm. (**G**) The surface areas of transfected cells were measured using ImageJ (n = 20, **P < 0.01, *t*-test). (**H**) The mRNA expression of the hypertrophic marker Nppa *was analyzed* by qPCR in the transfected mouse primary cardiomyocytes.

Considering a previous report showing activation and translocation of Ck2α upon hypertrophic stimuli (9), we investigated this pathway with respect to Dpf3a and examined whether hypertrophic stimuli induce phosphorylation of DPF3a mediated by Ck2α. We induced hypertrophic stress in mouse HL1, rat H9C2 cells as well as hiPSC-CMs using 10 μM phenylephrine (PE). The expression of *Nppa*, a marker of hypertrophy, increased in HL1 and H9C2 cells upon PE treatment (Supplementary Figure S5A). PE stimulation significantly increased the mRNA expression (Supplementary Figure S5A) and the phosphorylation of Dpf3a in both HL1 and H9C2 cells (Supplementary Figure S5B). During differentiation of hiPSC-CMs, the protein level of pDPF3a increased on day 5 and remained stable thereafter (Supplementary Figure S5C). In line with another publication (34), hiPSC-CMs did not respond to PE with unchanged *NPPA* expression (Figure 3A). However, endothelin 1 (ET-1) treatment successfully increased *NPPA* and *DPF3a* expression in hiPSC-CMs (Figure 3A). DPF3a phosphorylation was significantly increased upon ET-1 stimulation (Figure 3B). Moreover, we observed much higher levels of DPF3a phosphorylation in hypertrophic left ventricle of patients with HCM and AS compared to NH hearts (Figure 3C). In addition, many more pDPF3a positive cells were found in the hypertrophic right ventricle of TOF hearts compared to NH hearts (Figure 3D). Furthermore, knockdown of DPF3 in hiPSC-CMs decreased the increment of *NPPA* expression in the ET-1 situation (Figure 3E), suggesting that ET-1 induced hypertrophy can be buffered by the abolition of DPF3. To further investigate whether S348 phosphorylation is required for hypertrophy, mouse primary cardiomyocytes were transfected with Flag-tagged WT DPF3a or S348A mutant constructs (Figure 3F). Non-transfected cells were used as controls and stained for cell size measurements. Overexpression of DPF3a WT increased cell size and *Nppa* expression, whereas cell size changes and *Nppa* expression alterations could not be observed in those transfected with the S348A mutant constructs (Figure 3, G and H), indicating that the phosphorylation of S348 is required for the hypertrophic response.

### DPF3a interacts with the transcription repressors HEY

To identify downstream targets of DPF3a, we performed protein binding microarray (PBM) and ChIP-seq analysis. In the PBM experiments, a purified GST-tagged DPF3a or DPF3b protein was applied to a ‘universal’ PBM representing all 10 bp double-stranded DNA (dsDNA) sequences (35). Neither DPF3a nor DPF3b exhibited sequence-specific binding to dsDNA (data not shown). However, previously we found several hundred sites of indirect DNA binding using ChIP-chip. For DPF3b, we could show that this binding is mediated by specific histone marks (20). Focusing on pDPF3a, we now identified approximately 800 binding sites in ChIP-seq experiments using undifferentiated and differentiated C2C12 cells (data not shown). Using a pattern matching approach, we found that the E-box motif, which represents the favored binding motif of basic helix-loop-helix (bHLH) transcription factors, is highly enriched in pDPF3a target sites (Supplementary Table S6).

**Figure 4.**
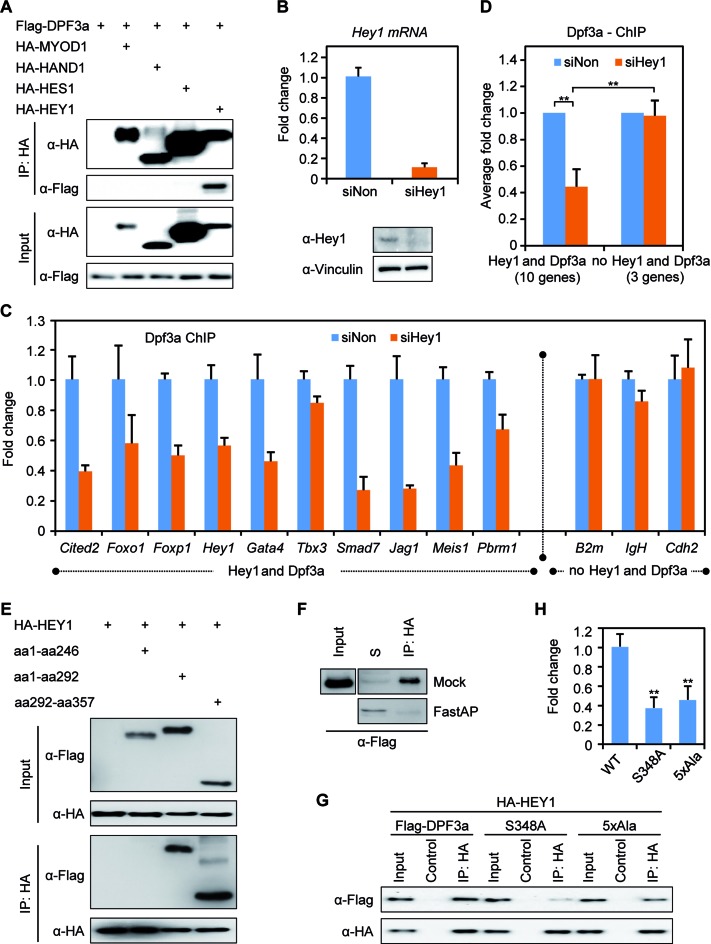
HEY protein links DPF3a to DNA. (**A**) DPF3a specifically interacts with HEY1. HEK293 cells were transiently transfected with Flag-tagged DPF3a and with HA-tagged MYOD1, HAND1, HES1 or HEY1. Cell lysates were incubated with an anti-HA matrix or anti-Flag matrix to pull down HA-tagged or Flag-tagged protein together with interacting proteins, which were further probed with anti-Flag or anti-HA antibody in immunoblots. (**B**) C2C12 cells were transfected with Hey1 siRNA for 48 h and knockdown efficiency of *Hey1* in C2C12 cells was analyzed by qPCR and immunoblotting. (**C** and **D**) Dpf3a ChIP experiments were performed and Dpf3a enrichment at selected promoters in siHey1 knockdown was compared to siNon control. Binding of Dpf3a was quantified by qPCR (triplicate experiments, **P < 0.01, *t*-test). (**E**) The interaction of DPF3a and HEY1 is mediated via the half PHD finger. Co-immunoprecipitation (CoIP) of HA-tagged HEY1 with different Flag-tagged DPF3a constructs (WT, aa1–246, aa1–292 and aa292–357) from lysates of transiently transfected HEK293 cells. (**F**) CoIP of Flag-tagged DPF3a and HA-tagged HEY1 followed by de-phosphorylation assay using FastAP Alkaline phosphatase. S: supernatant. (**G**) Phosphorylation of serine 348 of DPF3a is critical for the interaction with HEY1. CoIP of HA-tagged HEY1 with Flag-tagged DPF3a mutants (WT, S348A, 5xAla). (**H**) Quantification of the DPF3a and HEY1 interaction using Image Lab 5.0 (triplicate experiments, **P < 0.01, *t*-test).

bHLH transcription factors, such as HAND1/2, MYOD1, HES1 and HEY1/2, are important for embryonic development with respect to cardiac and muscle development. In order to study the interaction of DPF3a with these bHLH transcription factors (MYOD1, HAND1, HES1 and HEY1), we performed CoIP analysis with lysates of transiently transfected HEK293 cells. We observed a specific interaction between DPF3a and HEY1, the hairy and enhancer of split-related family of bHLH-type transcriptional repressors (Figure 4A). HEY genes redundantly function during mouse embryonic cardiovascular development, neurogenesis and somitogenesis (36), and thus match the expression and proposed function of DPF3a. Finally, we found that all proteins of the HEY family, namely HEY1, HEY2 and HEYL, specifically interact with DPF3a (Supplementary Figure S6A). In the following study, we exclusively focus on HEY1 as an example to analyze the function of DPF3a-HEY interactions.

We further identified common targets of Dpf3a and Hey1 using ChIP-qPCR experiments in C2C12 cells (Supplementary Figure S7A), in which *Hey1* is highly expressed in contrast to almost no expression of *Hey2* and *Heyl* (Supplementary Figure S7B). These results indicate that Dpf3a might indirectly bind DNA via its interaction with the DNA-binding transcriptional repressor Hey1 in C2C12 cells. To confirm this hypothesis, we performed siRNA knockdown of *Hey1* in C2C12 cells, which resulted in a notable reduction of Hey1 expression on mRNA and protein level (Figure 4B). Inhibition of *Hey1* did not cause expression alterations of *Hey2* or *Heyl* (Supplementary Figure S6B). We checked Dpf3a binding in the Hey1 knockdown situation and found a striking reduction of Dpf3a enrichment at selected targets (Figure 4, C and D). In line with this, we also analyzed Hey1 enrichment at these targets in a Dpf3a knockdown (Supplementary Figure S7C) situation in C2C12 cells and observed no significant changes of Hey1-DNA binding (Supplementary Figure S7D and E). These results underline that Hey1 indeed serves as a linker between Dpf3a and DNA.

Since posttranslational modifications like phosphorylation can modulate the nature and strength of protein–protein interactions, we aimed to elucidate the protein domain of DPF3a required for the interaction with HEY1 and investigated whether this interaction depends on the phosphorylation state of DPF3a. We assumed that the DPF3a-HEY1 interaction is mediated by the DPF3a specific half PHD and/or C-terminus, as we did not observe an interaction between DPF3b and HEY1 (Supplementary Figure S6, B and C). Using CoIP analysis, we found that lacking the half PHD finger prevents the interaction of DPF3a with HEY1, confirming this hypothesis (Figure 4E).

Furthermore, we analyzed whether DPF3a phosphorylation affects its interaction with HEY1. We performed CoIP analysis of HEY1 with DPF3a followed by *in vitro* de-phosphorylation of the CoIP pellet using FastAP alkaline phosphatase. Immunoblotting analysis showed that de-phosphorylation of the DPF3a-HEY1 complex abolished their interaction (Figure 4F). Finally, we co-transfected HA-tagged HEY1 with Flag-tagged WT DPF3a, S348A or 5xAla mutant into HEK293 cells. Similar expression of WT, S348A and 5xAla DPF3a protein levels were detected (Figure 4G, lane 1, 4, 7). We found that WT DPF3a efficiently interacted with HEY1 (Figure 4G, lane 3), but the S348A and 5xAla mutants showed a strikingly decreased interaction (Figure 4G, lane 6, 9, Figure 4H). These results suggest that the S348 phosphorylation is critical for the DPF3a-HEY1 interaction mediated by the half PHD finger.

### DPF3a recruits BRG1 to genomic targets

**Figure 5.**
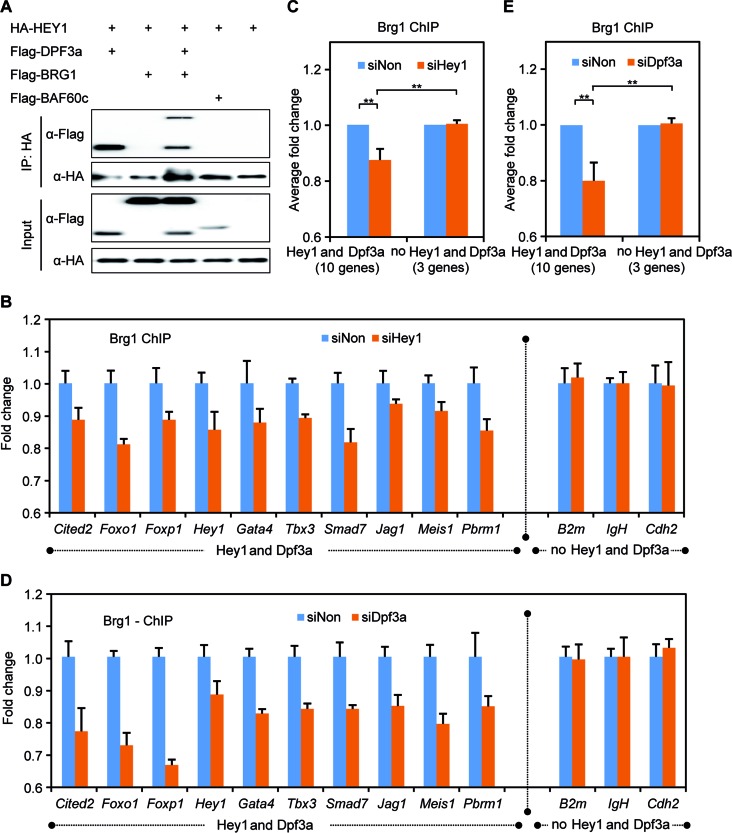
DPF3a recruits BRG1 to DNA via interaction with HEY. (**A**) HEY1 specifically interacts with DPF3a and associates with the BAF complex via this interaction. CoIP of HA-tagged HEY1 with Flag-tagged DPF3a, BAF60c and BRG1 constructs from lysates of transiently transfected HEK293 cells. (**B** and **C**) Brg1 ChIP experiments were performed and Brg1 enrichment at selected promoters in siHey1 knockdown was compared to siNon control. Binding of Brg1 was quantified by qPCR (triplicate experiments, **P < 0.01, *t*-test). (**D** and E) Brg1 enrichment at selected promoters in siHey1 knockdown was compared to siNon control. Binding of Brg1 was quantified by ChIP and real-time PCR (triplicate experiments, **P < 0.01, *t*-test).

Previous work has shown that both DPF3a and DPF3b are in a complex with the BAF chromatin remodeling complex and interact with other BAF components like BRG1 and BAF60c (20,37). To determine whether HEY1 interacts with any other BAF components or whether its interaction with DPF3a is independent of the BAF complex, we performed CoIP assays. We did not find any interaction between HEY1 and BRG1 or BAF60c (Figure 5A, lane 2, 4). However, HEY1, DPF3a and BRG1 formed a complex in the CoIP assay (Figure 5A, lane 3). Thus, our results suggest that HEY1 is associated with the BAF complex via DPF3a and the interaction between DPF3a and HEY1 is independent of the BAF complex.

To confirm this hypothesis, we performed ChIP-qPCR in order to identify common Brg1-Dpf3a-Hey1 targets in WT C2C12 and after Hey1 knockdown and found a complementary reduction of Brg1 binding to Dpf3a-Hey1 target sites (Figure 5, B and C). Moreover, we observed a reduction of Brg1 binding at respective targets sites in the Dpf3a knockdown condition (Figure 5, D and E). Taken together, these results indicate that the Dpf3a-Hey1 complex directs the BAF chromatin remodeling complex to specific genomic target sites.

### DPF3a releases HEY from the genomic targets

**Figure 6.**
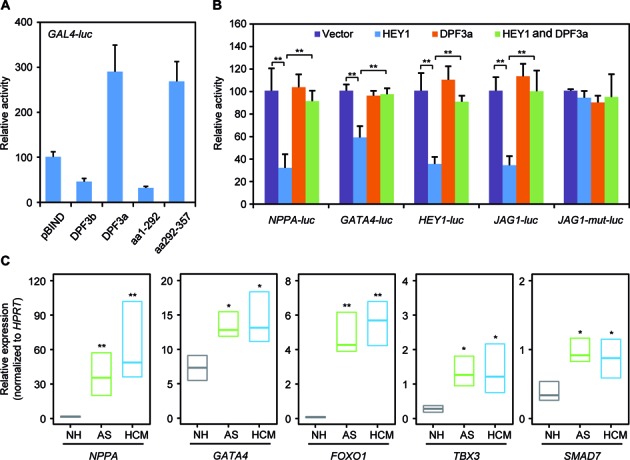
DPF3a releases HEY from the targets. (**A**) GAL4 trans-activation assays. HEK293 cells were co-transfected with an expression vector encoding DPF3 protein fragments fused to the GAL4 binding domain and a reporter vector containing the GAL4 promoter linked to the firefly luciferase gene. Luciferase activity was normalized to renilla luciferase activity. One representative experiment performed in triplicates is shown. (**B**) DPF3a significantly reduces HEY1 mediated repression. HEK293 cells were transiently transfected with *NPPA, GATA4, HEY1, JAG1* and *JAG1-mut* reporter constructs in the presence of empty control vector alone, *HEY1* expression vector, *DPF3a* expression vector or *HEY1* and *DPF3a* in combination. The results represent the average of three independent experiments (**P < 0.01, *t*-test). (**C**) Expression analysis of *FOXO1, GATA4, TBX3* and *SMAD7* in AS, HCM and NH using qPCR. Results represent median expression levels with 25% and 75% quartiles. Statistically significant differences were analyzed using a two-sided Student *t*-test (*P < 0.05, **P < 0.01).

To examine the role of DPF3a in transcriptional activation, HEK293 cells were co-transfected with an expression vector encoding DPF3 protein fragments fused to the GAL4 binding domain and a reporter vector containing the GAL4 promoter linked to the firefly luciferase gene. Only DPF3a, but not DPF3b, showed transactivation activity, which is transmitted by the DPF3a specific C-terminus (aa291–357) (Figure 6A). This underlines a role of DPF3a in the transcriptional activation.

Previous studies showed that HEY proteins function as direct transcriptional repressors and a number of HEY target genes involved in cardiac hypertrophy have been identified previously (38–41). In our reporter gene assays, HEY1 significantly repressed the promoter activity of its target genes like *NPPA, GATA4, HEY1* and *JAG1* (Figure 6B). DPF3a alone had no impact on the promoter activity of these target genes. Subsequently, co-transfection of reporter constructs with HEY1 and DPF3a expression vectors revealed no suppression of transcriptional activities of these targets. DPF3a notably reduced the repression of HEY1 targets (Figure 6B), suggesting that DPF3a might release HEY1 from DNA via a protein–protein interaction. In addition to this, mutation of the HEY1 binding E-box element (gggCACGGGtca to gggCAtca) in the *JAG1* promoter abolished the repression by HEY1.

Furthermore, we analyzed the expression of *HEY1* and *HEY2* genes in hypertrophic hearts of patients with AS and HCM, in which *DPF3a* was found to be up-regulated. We did not observe any obvious alteration of the expression of the HEY genes (Supplementary Figure S8). However, DPF3a-HEY1 targets, including the hypertrophic marker *NPPA* as well as *FOXO1* (forkhead box O1), *GATA4* (GATA binding protein 4), *TBX3* (T-box 3) and *SMAD7* (SMAD family member 7), were found to be significantly up-regulated (Figure 6C).

### Model of the CK2-DPF3a-HEY-BRG1 pathway

**Figure 7.**
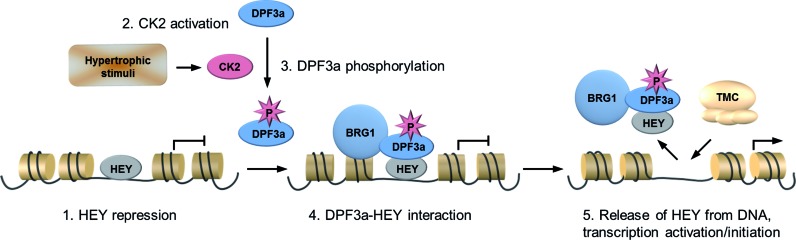
Illustration of pDPF3a mediated regulation of HEY-target genes. Hypertrophic signals stimulate CK2, which phosphorylates DPF3a at S348. Phosphorylation of DPF3a enhances its ability to bind the transcription repressors HEY. Subsequently, DPF3a releases HEY from the genomic targets and the transcription is then activated by the transcriptional machinery complex (TMC).

Finally, we developed a model summarizing our findings (Figure 7). Upon hypertrophic stimuli, the protein kinase CK2 phosphorylates DPF3a at the highly conserved amino acid S348 at its C-terminus. pDPF3a binds and releases the transcriptional repressors HEY and recruits BRG1 to respective targets. Consequently, DPF3a initiates transcription of fetal genes and promotes pathological cardiac hypertrophy.

## DISCUSSION

We present a novel pathway driving pathological cardiac hypertrophy in response to hypertrophic stimuli. This pathway is triggered by kinase activation leading to phosphorylation of a BAF chromatin remodeling factor and consequently triggering the release of DNA-binding transcriptional repressors. In the past, reports have demonstrated the impact of individual components, namely CK2, BRG1 and HEY, involved in the process of cardiac hypertrophy. However, their connection through the chromatin remodeler DPF3a had not yet been elucidated.

The SWI/SNF chromatin remodeling complexes are recruited to numerous genomic target sites by sequence-specific transcription factors and histone-binding epigenetic regulators (42). Each SWI/SNF complex utilizes either BRG1 or BRM as alternative subunits with DNA-dependent ATPase activity. The non-catalytic subunits of SWI/SNF are often referred to as BAFs (BRG1 or BRM associated factors). With respect to cardiac diseases, several BAFs (BAF60c, BAF45c2/DPF3b) have been identified to play a role in the development of congenital structural heart diseases. BAF60c mediates its function by the interaction with DNA-binding transcription factors such as TBX5, GATA4, MYOD or SIX4, which are essential for cardiac and muscle development (16,18,43). Another mode of functional transition is the binding to specific histone modifications, as in case of DPF3b (20). Either way, these BAFs promote gene transcription through their association with BRG1, which is highly expressed in the developing heart. In contrast, in the adult heart BRM represents the prominent catalytic unit; however, upon hypertrophic stimuli cardiomyocytes switch to a fetal-like state and BRG1 is reactivated as the catalytic unit (14,44). It had been shown that BRG1 is up-regulated in patients with hypertrophic cardiomyopathy and, moreover, forms a complex with PARPs and several HDACs to induce the fetal gene program in transverse aortic constriction-banded mice (14). In line with this, Brg1 null mice show significantly reduced hypertrophic response upon TAC-binding compared to WT mice (14). However, it was unclear how BRG1 is targeted to genes of the fetal gene program in this setting. Our data suggest that DPF3a functions as the mediator to direct BRG1 to fetal genes that are repressed by HEY proteins in a WT adult stage but are re-expressed upon hypertrophic stimuli. Moreover, we show that the binding of DPF3a to HEY proteins is significantly enhanced by phosphorylation of S348, located in an evolutionary conserved and DPF3a-specific C-terminal domain. The phosphorylation level of DPF3a is significantly higher in hypertrophic states (e.g. ET-1 treated hiPSC-CMs or hypertrophic TOF hearts). This phosphorylation of DPF3a is mediated by CK2. Previously it had been shown that signaling of hypertrophic stimuli (e.g. PE) can be mediated by CK2, involving its two catalytic subunits CK2α and CK2α’. CK2α was shown to phosphorylate and thereby activate HDAC2, which results in the repression of anti-hypertrophic genes (9). CK2α’ was shown to induce cardiac hypertrophy via interacting with the anti-hypertrophic cell cycle regulator p27 (7). Insights into the down-stream mediators, namely chromatin or transcription factors, and a potential role of DPF3a await elucidation.

In general, protein phosphorylation is a key event in regulating protein localization, activity and protein–protein interactions (45,46). We found that pDPF3a is predominately located in the nucleus. It had previously been shown that CK2α translocates from the cytoplasm to the nucleus (9), where it is suggested to phosphorylate DPF3a. However, this needs further investigation, as a non-pDPF3a specific antibody is currently lacking. Moreover, the conformational change of DPF3a upon phosphorylation awaits further analysis. Frequently, phosphorylation occurs within an interaction domain and thereby directly affects the binding energy (45). However, the interaction of DPF3a with HEY proteins is mediated by its half PHD finger, whereas S348 phosphorylation is located in the structurally unsolved neighboring C-terminus. It is very likely that a conformational change through allosteric mechanisms affects the DPF3a-HEY interaction.

In patients with pathological cardiac hypertrophy suffering from aortic stenosis or hypertrophic cardiomyopathy, we observed a significant up-regulation of common DPF3a and HEY targets, such as *GATA4, FOXO1, SMAD7* and *TBX3*. These genes play well-known roles in cardiac development and disease (47–50). For instance, GATA4 regulates cardiac fetal genes (51) and stress-induced cardiac hypertrophy *in vivo* (52). Activation of FOXO1 is an important mediator of cardiac hypertrophy (53).

In the future, it will be of interest to study the development of pathological hypertrophy in a respective mouse model. So far, we have generated a Dpf3^tm1sper^ knockout mouse, which is viable and fertile (data not shown), and can undergo TAC-banding in order to study its hypertrophic response. In this study, we observed that knockdown of DPF3 buffered the ET-1 induced hypertrophy in hiPSC-CMs. It might be that a reduction of hypertrophy in the knockout compared to the WT mouse will be observed. An essential aspect for these experiments is that, within the D4 protein family, the protein domains of Dpf3a involved in the novel pathway described here are unique for Dpf3a. In contrast, all Dpf3b domains are highly conserved within the D4 family, consisting of neural expressed Dpf1 and ubiquitously expressed Dpf2. Thus, other D4 family members most likely balance the physiological role of Dpf3 during development.

A number of HEY mouse models have been established (40,54). Interestingly, *HEY2* knockout mice on a mixed genetic background develop cardiomyopathy with cardiac hypertrophy (55). Moreover, overexpression of *HEY2* in the myocardium prevents PE-induced cardiac hypertrophy *in vivo* and *in vitro* (56). The anti-hypertrophic function of HEY2 is most likely mediated by the inhibition of fetal genes like *Nppa* and *Gata4*. Our model suggests that DPF3a induces pathological cardiac hypertrophy by the release of HEY from these genes. It had been shown that members of the HEY protein family (HEY1, HEY2, HEYL) share genomic targets and perform similar functions (41). Moreover, we show that all three HEY proteins physically bind to DPF3a. Thus, DPF3a can be considered as a cofactor of HEY proteins in order to regulate cardiac gene expression.

Taken together, we provide evidence for a novel pathway involved in the development of pathological cardiac hypertrophy. The latter can occur primarily in cases of hypertrophic cardiomyopathy or Tetralogy of Fallot or secondary, due to pressure overload like in cases of aortic stenosis or hypertension. Pathological cardiac hypertrophy leads to remodeling of the ventricular chambers and eventually heart failure. Thus, pharmacological inhibition of hypertrophic cardiomyocyte growth has been a long-term therapeutic goal for the treatment of heart failure. Future studies are needed to explore the potential of the pathway presented to be targeted for pharmaceutical interventions.

## Supplementary Material

SUPPLEMENTARY DATA
