# Supplementary material for: Phosphorylation of the chromatin remodeling factor DPF3a induces cardiac hypertrophy through releasing HEY repressors from DNA
Source: Nucleic Acids Res. 2015 Nov 17;44(6):2538–53. doi: 10.1093/nar/gkv1244 (PMC4824069; doi:10.1093/nar/gkv1244)
Supplement: SUPPLEMENTARY DATA [file supp_44_6_2538__index.html]

Phosphorylation of the chromatin remodeling factor DPF3a induces cardiac hypertrophy through releasing HEY repressors from DNA — Phosphorylation of the chromatin remodeling factor DPF3a induces cardiac hypertrophy through releasing HEY repressors from DNA — Phosphorylation of the chromatin remodeling factor DPF3a induces cardiac hypertrophy through releasing HEY repressors from DNA — SUPPLEMENTARY DATA 

# Phosphorylation of the chromatin remodeling factor DPF3a induces cardiac hypertrophy through releasing HEY repressors from DNA

## SUPPLEMENTARY DATA

- SUPPLEMENTARY DATA
