# Supplementary material for: Phosphorylation of the chromatin remodeling factor DPF3a induces cardiac hypertrophy through releasing HEY repressors from DNA
Source: Nucleic Acids Res. 2015 Nov 17;44(6):2538–53. doi: 10.1093/nar/gkv1244 (PMC4824069; doi:10.1093/nar/gkv1244)
Supplement: SUPPLEMENTARY DATA [file supp_gkv1244_NAR_gkv1244_Supplementary_data.pdf]

Supplementary Data to:

Phosphorylation of the chromatin remodeling factor DPF3a induces cardiac hypertrophy through releasing HEY repressors from DNA

Huanhuan Cui[1,2,3], Jenny Schlesinger[1,3], Sophia Schoenhals[1,2], Martje Tönjes[3], Ilona Dunkel[3], David Meierhofer[4], Elena Cano[1], Kerstin Schulz[1], Michael F. Berger[5,6], Timm Haack[7], Salim Abdelilah-Seyfried[7,8], Martha L. Bulyk[5,6,9], Sascha Sauer[4,10], Silke R. Sperling[1,2,3,11*]

[1] Department of Cardiovascular Genetics, Experimental and Clinical Research Center, Charité - Universitätsmedizin Berlin and Max Delbrück Center for Molecular Medicine, 13125 Berlin, Germany

[2] Department of Biology, Chemistry, and Pharmacy, Freie Universität Berlin, 14195 Berlin, Germany

[3] Group of Cardiovascular Genetics, Department of Vertebrate Genomics, Max Planck Institute for Molecular Genetics, 14195 Berlin, Germany

[4] Max Planck Institute for Molecular Genetics, 14195 Berlin, Germany

[5] Division of Genetics, Department of Medicine, Brigham and Women's Hospital and Harvard Medical School, Boston, MA 02115, USA

[6] Committee on Higher Degrees in Biophysics, Harvard University, Cambridge, MA 02138, USA

[7] Hannover Medical School, Institute of Molecular Biology, Carl-Neuberg Str. 1, D-30625 Hannover, Germany

[8] Potsdam University, Institute of Biochemistry and Biology, Department of Animal Physiology, Karl-Liebknecht Str. 24-25, 14476 Potsdam-Golm, Germany

[9] Department of Pathology, Brigham and Women's Hospital and Harvard Medical School, Boston, MA 02115, USA

[10] CU Systems Medicine, University of Würzburg, 97080 Würzburg, Germany

[11] DZHK (German Center for Cardiovascular Research), partner site Berlin, Berlin, Germany

* To whom correspondence should be addressed. Tel: +4930450540123; Fax: +4930450540159; Email: silke.sperling@charite.de

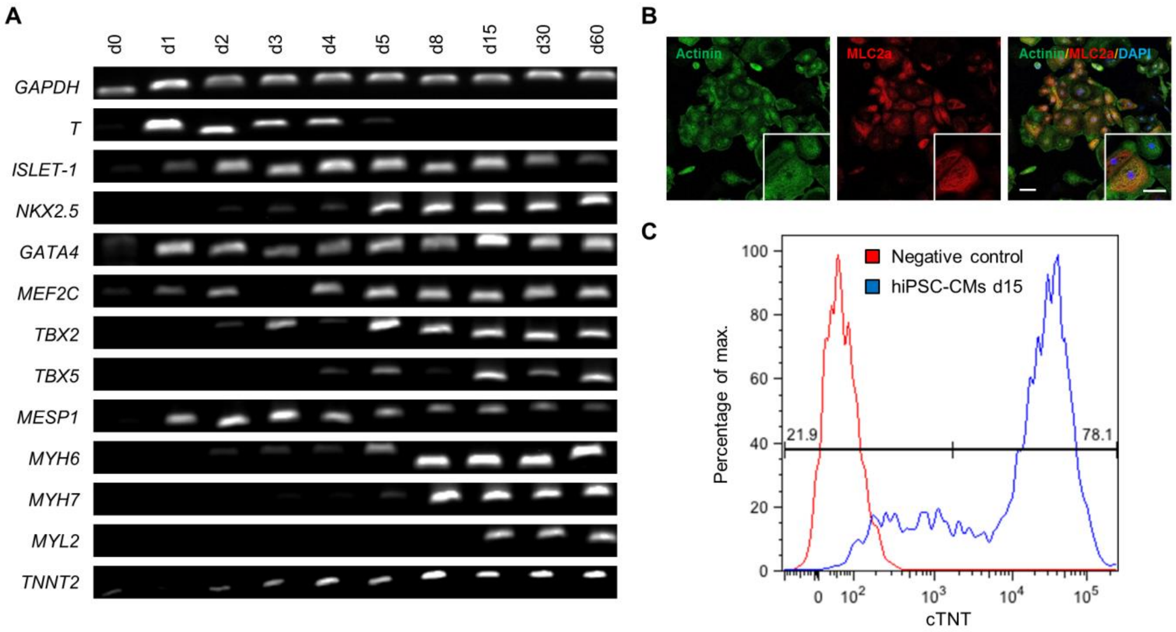

Supplementary Figure S1. Characterization of successful differentiation of human-induced pluripotent stem cells to cardiomyocytes.

(A) Analysis of mesodermal and cardiac gene expression during differentiation from day 0 to day 60.

(B) Immunostaining for characteristic cardiac markers. Scale bars indicate 50 and 20 µM, respectively.

(C) Cardiac Troponin T expression was analyzed at day 15 by flow cytometry.

```
MATVIHNPLK ALGDQFYKEA IEHCRSYNSR LCAERSVRLP FLDSQTGVAQ NNCYIWMEKR  60
HRGPGLAPGQ LYTYPARCWR KKRRLHPPED PKLRLLEIKP EVELPLKKDG FTSESTTLEA 120
LLRGEGVEKK VDAREEESIQ EIQRVLENDE NVEEGNEEED LEEDIPKRKN RTRGRARGSA 180
GGRRRHDAAS QEDHDKPYVC DICGKRYKNR PGLSYHYAHT HLASEEGDEA QDQETRSPPN 240
HRNENHRPQK GPDGTVIPNN YCDFCLGGSN MNKKSGRPEE LVSCADCGRS AHLGGEGRKE 300
KEAAAAARTT EDLFGSTSES DTSTFHGFDE DDLEEPRSCR GRRSGRGSPT ADKKGSC     357
```

Netphos 2.0 score > 0.5

CK2 KinasePhos 2.0 score > 0.5

Supplementary Figure S2. Phosphorylation prediction of DPF3a.

Prediction of phosphorylation sites within the human DPF3a sequence (NP_036206) using the prediction tools Netphos 2.0 and KinasePhos 2.0.

```
M.musculus          MATVIHNPLKALGDQFYKEAIEHCRSYNSRLCAERSVRLPFLDSQTGVAQNNCYIWMEKR 60
R.norvegicus        MATVIHNPLKALGDQFYKEAIEHCRSYNSRLCAERSVRLPFLDSQTGVAQNNCYIWMEKR 60
H.sapiens           MATVIHNPLKALGDQFYKEAIEHCRSYNSRLCAERSVRLPFLDSQTGVAQNNCYIWMEKR 60
M.mulatta           MATVIHNPLKALGDQFYKEAIEHCRSYNSRLCAERSVRLPFLDSQTGVAQNNCYIWMEKR 60
G.gallus1           MATVIHNPLKALGDQFYKEAIEHCRSYNSRLCAERSVRLPFLDSQTGVAQNNCYIWMEKR 60
G.gallus2           MATVIHNPLKALGDQFYKEAIEHCRSYNSRLCAERSVRLPFLDSQTGVAQNNCYIWMEKR 60
                    ************************************************************

M.musculus          HRGPGLAPGQLYTYPARCWRKKRRLHPPEDPKLRLLEIKPEVELPLKKDGFTSESTTLEA 120
R.norvegicus        HRGPGLAPGQLYTYPARCWRKKRRLHPPEDPKLRLLEIKPEVELPLKKDGFTSESTTLEA 120
H.sapiens           HRGPGLAPGQLYTYPARCWRKKRRLHPPEDPKLRLLEIKPEVELPLKKDGFTSESTTLEA 120
M.mulatta           HRGPGLAPGQLYTYPARCWRKKRRLHPPEDPKLRLLEIKPEVELPLKKDGFTSESTTLEA 120
G.gallus1           HRGPGLAPGQLYTYPARCWRKKRRLHPPEDSRLKLLEIKPEVDLPLKKDGFTSESTTLEA 120
G.gallus2           HRGPGLAPGQLYTYPARCWRKKRRLHPPEDSRLKLLEIKPEVDLPLKKDGFTSESTTLEA 120
                    ******************************.:*:********:*****************

M.musculus          LLRGEGVEKKVDAREEESIQEIQRVLENDENVEEGNEEEDLEEDVPKRKNRTRGR----- 175
R.norvegicus        LLRGEGVEKKVDAREEESIQEIQRVLENDENVEEGNEEEDLEEDIPKRKNRTRGR----- 175
H.sapiens           LLRGEGVEKKVDAREEESIQEIQRVLENDENVEEGNEEEDLEEDIPKRKNRTRGR----- 175
M.mulatta           LLRGEGVEKKVDAREEESIQEIQRVLENDENVEEGNEEEDLEEDIPKRKNRTRGR----- 175
G.gallus1           LLRGEGIEKKMDTKEEDPIQEIQRVLENDENADEVNEEEDLEEDIPKRKNRPRGR----- 175
G.gallus2           LLRGEGIEKKMDTKEEDPIQEIQRVLENDENADEVNEEEDLEEDIPKRKNRPRGRPKTPT 180
                    ******:***:*::**:.*************.:* *********:******.***

M.musculus          --------ARGSAGGRRRHDAASQEDHDKPYVCDICGKRYKNRPGLSYHYAHTHLASEEG 227
R.norvegicus        --------ARGSAGGRRRHDAASQEDHDKPYVCDICGKRYKNRPGLSYHYAHTHLASEEG 227
H.sapiens           --------ARGSAGGRRRHDAASQEDHDKPYVCDICGKRYKNRPGLSYHYAHTHLASEEG 227
M.mulatta           --------ARGSAGGRRRHDAASQEDHDKPYVCDICGKRYKNRPGLSYHYAHTHLASEEG 227
G.gallus1           --------ARGSGGGRRRNDAASQDDHDKPYVCDICGKRYKNRPGLSYHYAHTHLASEEG 227
G.gallus2           WKKIFQKNARGSGGGRRRNDAASQDDHDKPYVCDICGKRYKNRPGLSYHYAHTHLASEEG 240
                            ****.*****:*****:***********************************

M.musculus          DEAQDQETRSPPNHRNENHRPQKGPDGTVIPNNYCDFCLGGSNMNKKSGRPEELVSCADC 287
R.norvegicus        DEAQDQETRSPPNHRNENHRPQKGPDGTVIPNNYCDFCLGGSNMNKKSGRPEELVSCADC 287
H.sapiens           DEAQDQETRSPPNHRNENHRPQKGPDGTVIPNNYCDFCLGGSNMNKKSGRPEELVSCADC 287
M.mulatta           DEAQDQETRSPPNHRNENHRPQKGPDGTVIPNNYCDFCLGGSNMNKKSGRPEELVSCADC 287
G.gallus1           DEAREQETRSSPVHRNENHKPQKGPDGVIIPNNYCDFCLGGSNMNKKSGRPEELVSCSDC 287
G.gallus2           DEAREQETRSSPVHRNENHKPQKGPDGVIIPNNYCDFCLGGSNMNKKSGRPEELVSCSDC 300
                    ***::*****.* ******:*******.:****************************:**

M.musculus          GRSAHLGGEGRKEKEAAAAARTTEDLFGSTSESDTSTFYGFDEDDLEEPRSCRGRRSGRG 347
R.norvegicus        GRSAHLGGEGRKEKEAAAAARTTEDLFGSTSESDTSTFYGFDEDDLEEPRSCRGRRSGRG 347
H.sapiens           GRSAHLGGEGRKEKEAAAAARTTEDLFGSTSESDTSTFHGFDEDDLEEPRSCRGRRSGRG 347
M.mulatta           GRSAHLGGEGRKEKEAAAAARTTEDLFGSTSESDTSTFHGFDEDDLEEPRSCRGRRSGRG 347
G.gallus1           GRSAHLGREGRRDE--AAPTRTTEDLFGSTSESDTSTFHGFDEDDAEEPLSSRGGGCGGS 345
G.gallus2           GRSAHLGREGRRDE--AAPTRTTEDLFGSTSESDTSTFHGFDEDDAEEPLSSRGGGCGGS 358
                    ******* ***:::  **.:******************:****** *** *.**  .* .

M.musculus          SPTADKKGSC 357
R.norvegicus        SPTADKKSSC 357
H.sapiens           SPTADKKGSC 357
M.mulatta           SPTADKKGSC 357
G.gallus1           SPSADKKGGC 355
G.gallus2           SPSADKKGGC 368
                    **:****..*
```

Supplementary Figure S3. Conservation analysis of DPF3a.

Protein sequence conservation analysis of DPF3a using ClustalW2 in *H. sapiens* (NP_036206), *M. mulatta* (AFE80282), *M. musculus* (NP_001254555), *R. norvegicus* (NP_001178747) and *G. gallus* (AAK51969.1 and AAK51970).

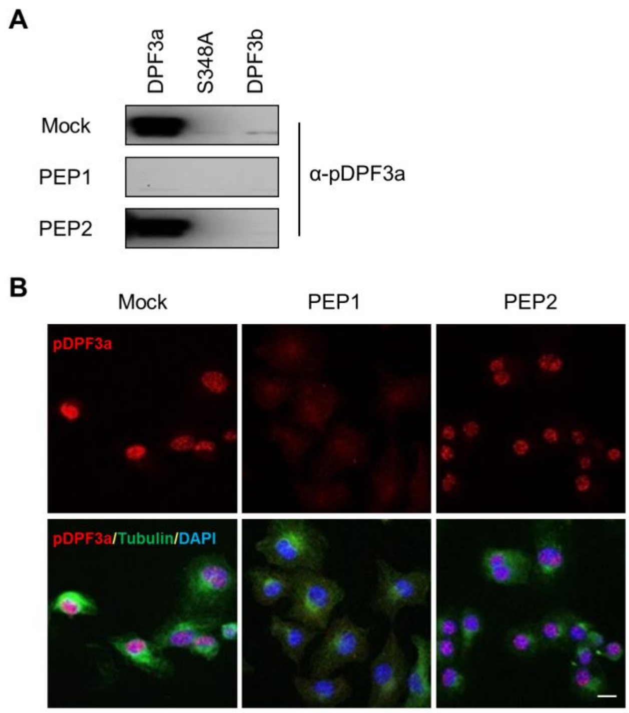

Supplementary Figure S4. Specificity of anti-pDPF3a.

(A) Flag-tagged DPF3a WT, S348A, and DPF3b from lysates of transiently transfected HEK293 cells were electrophoresed on a SDS-polyacrylamide gel and probed using the anti-pDPF3a antibody, pre-incubated with either PEP1 or PEP2 control peptide. PEP1: GRRSGRGS$^{(P)}$PTADKKGS; PEP2: GRRSGRGSPTADKKGS.

(B) HL1 cells were fixed and stained with antibody against pDPF3a (red) and antibody against Tubulin (green). Anti-pDPF3a antibody was pre-incubated with either PEP1 or PEP2 control peptide. Scale bar, 20 µm.

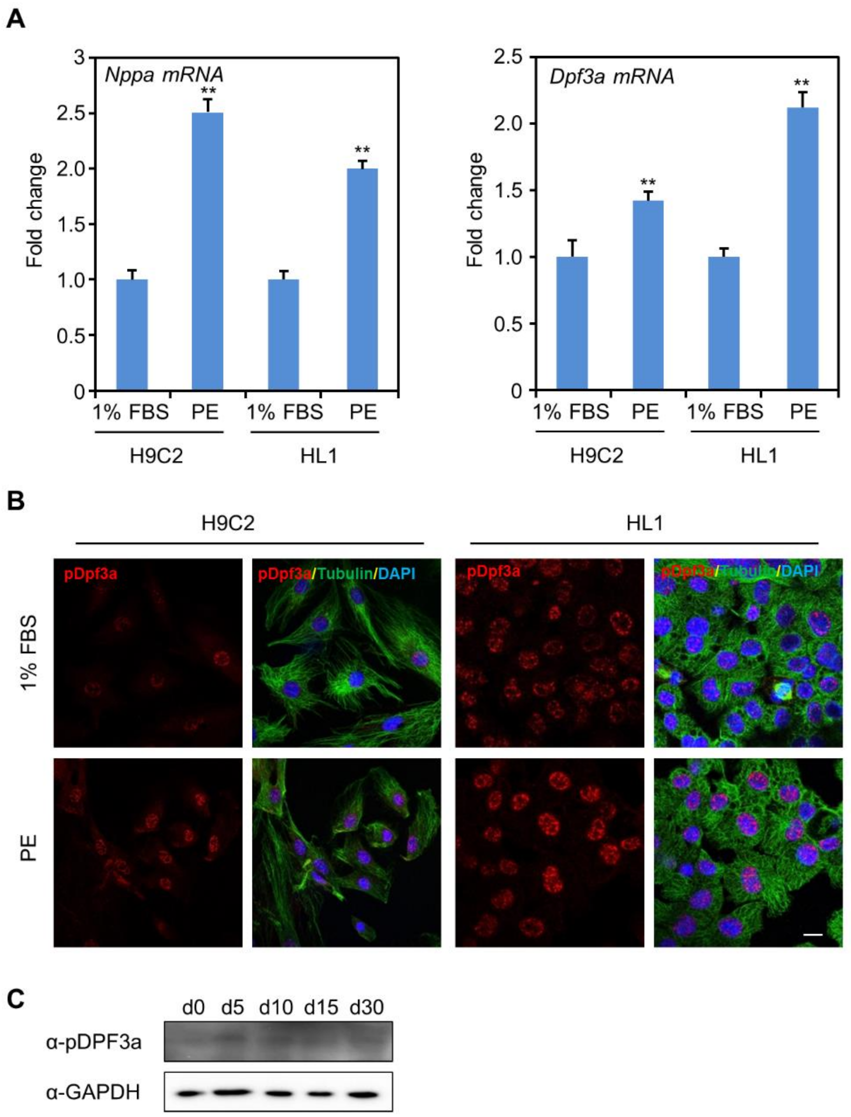

Supplementary Figure S5. DPF3a expression and phosphorylation in cardiac cells.

(A) *Nppa* and *Dpf3a* mRNA expression were analyzed in HL1 and H9C2 cardiomyocytes after phenylephrine (PE) treatment for 24 hours. 1% FBS was used as control.

(B) Nuclear localization of pDPF3a was increased by phenylephrine (PE) in HL1 and H9C2 cardiomyocytes. Red: pDPF3a; green: Tubulin. Scale bar, 20 µm.

(C) The protein level of phosphorylated DPF3a during differentiation of human-induced pluripotent stem cells to cardiomyocytes. Immunoblotting were performed on samples obtained from different day during cardiac differentiation.

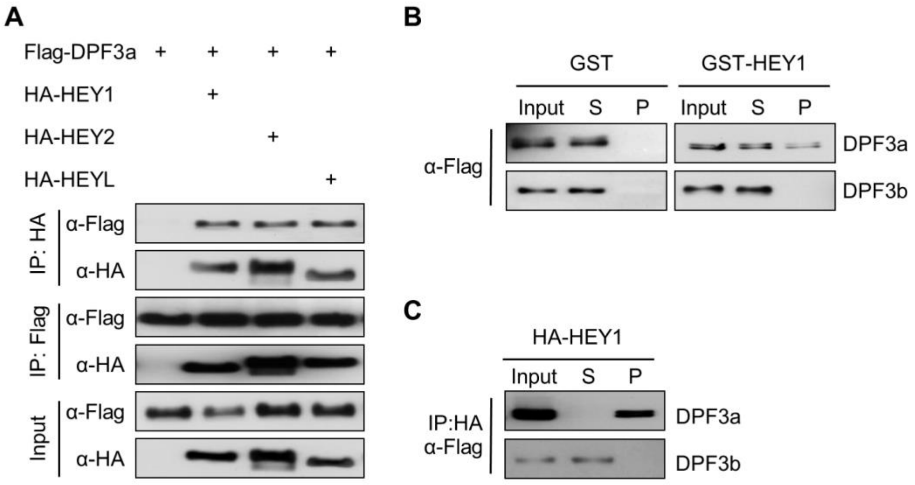

Supplementary Figure S6. Interactions between DPF3a/b and HEY proteins.

(A) DPF3a interacts with all HEY proteins. HEK293 cells were transiently transfected with Flag-tagged DPF3a and with HA-tagged HEY1, HEY2 or HEYL. Cell lysates were incubated with an anti-HA matrix to pull down HA-tagged protein together with interacting proteins, which were further probed with anti-Flag antibody in immunoblotting.

(B) GST pull-down with GST-HEY1 and Flag-DPF3a and DPF3b. GST vector was used as a negative control. S: supernatant, P: immunoprecipitation pellet.

(C) Co-immunoprecipitation of HA-tagged HEY1 with Flag-tagged DPF3a and DPF3b from lysates of transiently transfected HEK293 cells.

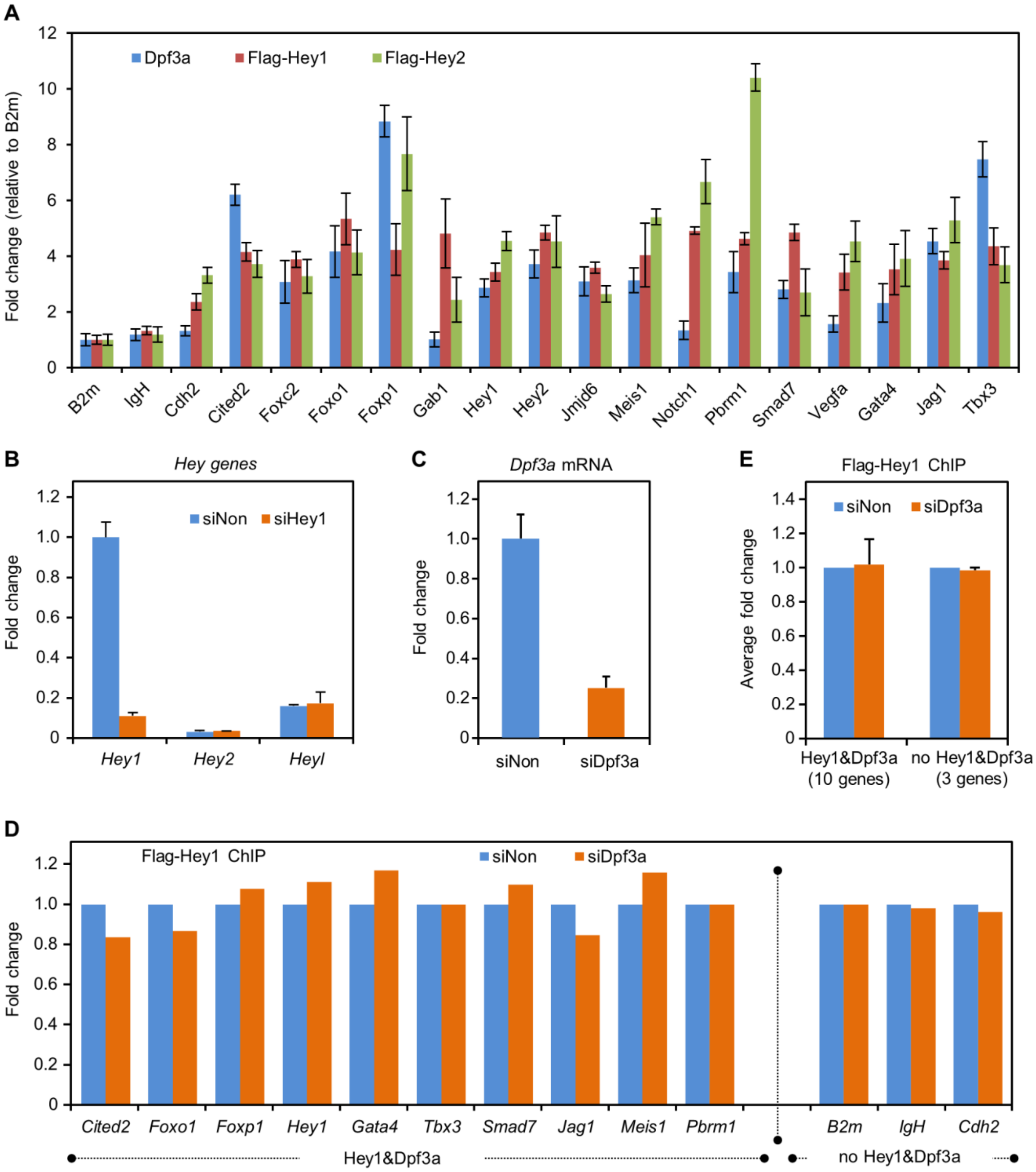

Supplementary Figure S7. ChIP-qPCR and siRNA knockdown in C2C12 cells

(A) Co-occurrence of Dpf3a, Hey1 and Hey2 binding. Dpf3a, Hey1 and Hey2 enrichment at selected promoters was analyzed using ChIP and qPCR.

(B) Expression levels of *Hey1*, *Hey2* and *Heyl* genes before and after siHey1 knockdown.

(C) Knockdown efficiency of *Dpf3a* in C2C12 cells was analyzed by qPCR.

(D-E) Hey1 enrichment at selected promoters in siDpf3a knockdown was compared to siNon control. Binding of Hey1 was quantified by ChIP and qPCR (triplicate experiments).

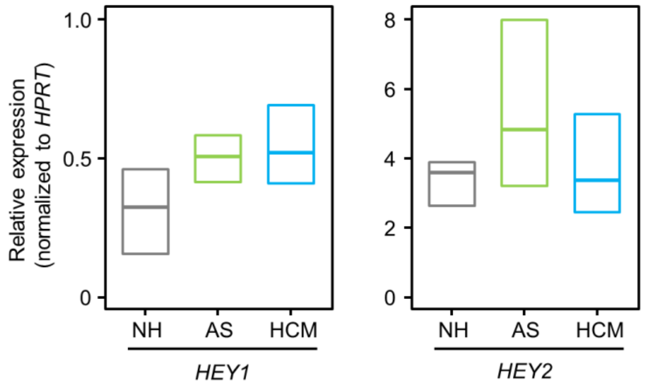

Supplementary Figure S8. *HEY1* and *HEY2* expression in human hearts.

Expression of *HEY1* and *HEY2* in aortic stenosis (AS), hypertrophic cardiomyopathy (HCM) and normal heart (NH) was analyzed using qPCR. Results represent median expression levels with 25% and 75% quartiles. Expression levels were analyzed by a two-sided Wilcoxon test and resulted in no significant findings between the analyzed groups.

Supplementary Table S1. Antibodies used in different experiments.

Antibodies used in chromatin immunoprecipitation (ChIP), Immunofluorescence (IF), Western blot (WB) and fluorescence-activated cell sorting (FACS), and their respective amounts/dilutions applied in the experiments are given.

**Primary antibodies**	**Company**	**Used amount/dilution**
Anti-SNF2β/BRG1, rabbit antiserum	Upstate (07-478)	ChIP: 5 µg
Anti-CK2 alpha, rabbit polyclonal	Abcam (ab10466)	WB: 1:500
Anti-Gapdh, mouse monoclonal	Ambion (AM4300)	WB: 1:5000
Anti-Flag, rabbit polyclonal	AnaSpec (29674)	IF: 1:500
Anti-Flag M2, mouse monoclonal	Sigma (F3165)	WB: 1:1000, IF: 1:400, ChIP: 2 µg
Anti-GST, rabbit polyclonal	Invitrogen (71-7500)	WB: 1:500
Anti-HA, rat monoclonal	Roche (11867423001)	WB: 1:2000
Anti-Hey1, rabbit polyclonal	Abcam (ab22614)	WB: 1:100
Anti-His HRP, mouse monoclonal	Macs Molecular (130-092-785)	WB: 1:5000
Anti-pDPF3a, rabbit polyclonal	Self-made	WB: 1:500, IF: 1:100, ChIP: 5 µg
Anti-Thiophosphate Ester, rabbit monoclonal	Epitomics (2686-1)	WB: 1:5000
Anti-Troponin I, goat polyclonal	HyTest (4T21/2)	IF: 1:200
Anti- Troponin T, mouse monoclonal	Lab Vision (MS-295)	FACS: 1:500
Anti- Mlc2a, mouse monoclonal	Synaptic Systems (311011)	IF: 1:200
Anti-Tubulin, mouse monoclonal	Sigma (T9026)	IF: 1:800
Anti-Actinin, mouse monoclonal	Sigma (A7811)	IF: 1:400
Anti-Vinculin, mouse monoclonal	Sigma (V9131)	WB: 1:25000
Normal Mouse IgG preimmuneserum	Santa Cruz Biotech (sc-2027)	ChIP: 5 µg
Normal Rabbit IgG preimmuneserum	Santa Cruz Biotech (sc-1237)	ChIP: 5 µg
**Secondary antibodies**		
Anti-mouse IgG (HRP)	Sigma(A0168)	WB: 1:10000
Anti-mouse IgG (Alexa Flour 488)	Invitrogen (A11029)	IF: 1:2000
Anti-rabbit IgG (Alexa Flour 568)	Invitrogen (A11036)	IF: 1:2000
Anti-rabbit IgG(Biotin)	Jackson lab(711-065-152)	IF: 1:500
Anti-goat IgG (Alexa Flour 568)	Invitrogen (A11057)	IF: 1:500
Anti-rabbit IgG (HRP)	Sigma (A2074)	WB: 1:10000
Anti-rat IgG (HRP)	Sigma (A9542)	WB: 1:20000

Supplementary Table S2. siRNAs used for knockdown experiments.

Sequences of siRNAs used in knockdown experiments.

Name	Target	Species	Company	Accession NO.	Target sequence
siDpf3a	Dpf3a	mouse	Invitrogen	NM_058212	TGACTCTGGTCATTGTTCTAGTTCT
siHey1	Hey1	mouse	Invitrogen	NM_010423	CGACGAGACCGAATCAATATT
siCk2a1	Ck2a1	mouse	Invitrogen	NM_007788	GATTATAGTTTGGATATGTTT
siNon	Synthetic	mouse	Invitrogen	Synthetic	Not provided
siFITC	Synthetic	mouse	Invitrogen	Synthetic	Not provided
siDPF3	DPF3	human	Qiagen	NM_012074	AAGGGAAATCAAAGAATCGA
					AAGAGGATATTCCCAAGCGAA
					AACAGACTCTCTGGGCAATTA
					AAGAAACGTATCAATACCCAT
siNON	Synthetic	human	Qingen	Synthetic	Not provided

Supplementary Table S3. Sequences of qPCR expression primers

Gene	Species	Accession Number	Forward Sequence (5' - 3')	Reverse Sequence (5' - 3')
Dpf3a	mouse	NM_058212	CAGACGGGACAGTCATTCCTAAT	CTCCCAAATGAGCAGAGCGT
Dpf3	mouse	NM_058212/NM_001267625	ACAACTGCTACATCTGGATGG	GTCGTAGTTTTGGGTCCTCTG
Hey1	mouse	NM_010423	TGAGCTGAGAAGGCTGGTAC	ACCCCAAACTCCGATAGTCC
Hey2	mouse	NM_013904	TGAGAAGACTAGTGCCAACAGC	TGGGCATCAAAGTAGCCTTTA
HeyL	mouse	NM_013905	CGTGGATCACTTGAAGATGC	CATTCCCGAAACCCAATACT
Ck2a1	mouse	NM_007788	GATAGCCAAGGTTCTGGGAA	CATCGCTTACGGGAGTGTC
Hprt	mouse	NM_013556	AAACAATGCAAACTTTGCTTTCC	GGTCCTTTTCACCAGCAAGCT
Dpf3a	rat	NM_001191818	AAGGCAGGAAGGAGAAGGAG	AGGCTCTTCCAAATCGTCCT
Nppa	rat	NM_012612	AGGGCTTCTTCCTCTTCCTG	TGTTGGACACCGCACTGTAT
Hprt	rat	NM_012583	TGCTGGTGAAAAGGACCTCTC	CCACTTTCGCTGATGACACAA
DPF3	human	NM_012074/NM_001280542	GGCTGCTGGAGATAAAACCTGA	TTCCTGGATGCTTTCCTCCTC
DPF3a	human	NM_012074	GACGATTTGGAAGAGCCTCG	GAGTCTGTTCCGTGGGTTTAGC
DPF3b	human	NM_001280542	CGAGGCTGTCAAGACCTACAAG	CGCAGAAGAGTAGCTGGTCATC
HEY1	human	NM_012258	ACGGGATGACCGTGGATCACCTGAAA	GCGTGCGCGTCAAAGTAAC
HEY2	human	NM_012259	ACGGGTTGAAGATGCTTCAGGCAACA	TCAGGTACCGCGCAACTTCT
NPPA	human	NM_006172	ACGGGTACAATGCCGTGTCCAACG	TTCTTCCAAATGGTCCAGCAA
TBX3	human	NM_005996	AAGGAGAATGGGACCTCTGA	CCTCGCTGGGACATAAATCT
FOXO1	human	NM_002015	CTGGCTCTCACAGCAATGAT	CACCATAGAATGCACATCCC
SMAD7	human	NM_001190821	CAGATTCCCAACTTCTTCTGG	CCCACTCTCGTCTTCTCCTC
HPRT	human	NM_000194	GGTGGAGATGATCTCTCAACTTTAA	AGGAAAGCAAAGTCTGCATTGTT
GAPDH	human	NM_002046.5	ACCCACTCCTCCACCTTTGA	TTGCTGTAGCCAAATTCGTTG
OCT4	human	NM_002701	AGTTTGTGCCAGGGTTTTTG	ACTTCACCTTCCCTCCAACC
NANOG	human	NM_024865	TTTGGAAGCTGCTGGGGAAG	GATGGGAGGAGGGGAGAGGA
T	human	NM_003181	CTCACCAATGAGATGATCGTGAC	AGGAAGGAGTACATGGCGTTG
ISLET-1	human	NM_002202	AGCAGCCCAATGACAAAACTAA	CGTGTCTCTCTGGACTGGCA
NKX2.5	human	NM_001166175	CACCTCAACAGCTCCCTGACT	ATCGCCGCCACAAACTCT
GATA4	human	NM_002052	ATCTCACTACGGGCACAGCA	TTTGAGGAGGGAAGAGGGAAG
TBX2	human	NM_005994	ACCCTGAGATGCCCAAAC	CAGTGACGGCGATGAAGT
MEF2C	human	NM_002397	TCAGGGACGAGAGAGAGAAGAAA	ACAGTCACACAGCACGCTCA
TBX5	human	NM_000192	CAGAAACTCAAGCTCACCAACAA	TGCTATAAACGCAGTCTCAGGAA
MYH6	human	NM_002471	GGTCATTGCTGAAACCGAGA	GCTCCTTGAGGTTGAAAAGCA
MESP1	human	NM_018670	ACGTGCTGGCTCTGTTG	GTCAGTTGTCCCTTGTCACTT
MYH7	human	NM_000257	GGATGTCTTCGTGCCTGATG	GGTCCTCCTTCACGGTCACT
MYL2	human	NM_000432	GGCGAGTGAACGTGAAAAAT	CAGCATTTCCCGAACGTAAT
MYL7	human	NM_021223	GAGGAGAATGGCCAGCAGGAA	GCGAACATCTGCTCCACCTCA
TNNT2	human	NM_000364	TTCACCAAAGATCTGCTCCTCGCT	TTATTACTGGTGTGGAGTGGGTGTGG

Supplementary Table S4. Primer sequences used for ChIP-qPCR in C2C12 cells

Gene	Accession NO.	Forward sequence (5' - 3')	Reverse sequence (5' - 3')
B2m	NM_009735	TGCCAAACCCTCTGTACTTCT	TTAGGCCTCTTTGCTTTACCA
Chd2	NM_001081345	CCGAGAAGTGTGTGTGTGTG	ACCTCCGTATCCTCCATCC
IgH	NG_005838	TGGTGGGGCTGGACAGAGTGT	GCCGATCAGAACCAGAACACC
Jag1	NM_013822	CAGCAAGCGAGCCCAGAG	TTTCAAAGTTCCCAGCAGAG
Smad7	NM_001042660	GCCAAAGGTCACCACCAT	TTGAGTTTCTTGAGCACCGA
Gata4	NM_008092	CTACCTGGCCAGCTCCAGT	CCTTGCACGTGACTCCCTTA
Gab1	NM_001301298	CCCAGAGGACGTCTCAGATT	CCGATCGAGTTCCTCTTCAG
Notch1	NM_008714	CGTTAGGCAGAGCAAGGG	GTGGTGTGCGTCAACGTC
Tbx3	NM_011535	TGACATAAACGCAGGACAGG	ACCAATTGTGTGGCTGCATA
Cited2	NM_010828	ATGGGCGAGCACATACACTA	ACCCATGAACTGGGAGTTGT
Foxc2	NM_013519	AGAAGAAGGATGTGCCCAAG	CCTCGCTCTTAACCACGACT
Foxo1	NM_019739	GTCGTACGCCGACCTCAT	GACAGGGTGAGCCTCTTCTC
Foxp1	NM_001197321	CAGGCGAGAAACGGAGAG	AGCTCTTCTCTGCGACACG
Vegfa	NM_001025250	TTGAATATCAAATCCCAGAGCA	GCAGCGACAAGGCAGACTAT
Hey1	NM_010423	GGGTGAGCTCTCTTCATGGT	GCAGTTAACTCCTCCTTGCC
Hey2	NM_013904	AGGAGGAGCAGCTACTGTCG	CAGCGTGGGAAAGAACCT
Meis1	NM_010789	AGACACTCCACAAATTCGCA	GCTGCAGCCTGTATTTGTGT
Pbrm1	NM_001081251	GCGCACACACATACACTGTC	CCCAGTGCTGCCGTAAAT

Supplementary Table S5. Demography of human subjects.

NH: normal heart, HCM: hypertrophic cardiomyopathy, AS: aortic stenosis

Clinical diagnosis	Age	Gender
NH	55	male
	43	male
	57	female
	37	female
AS	64	male
	68	male
	81	female
	68	female
HCM	50	male
	42	male
	53	female
	57	female
	51	female
	42	female
	50	female
	49	female

Supplementary Table S6. E-box motif identified within DPF3a peaks.

For ChIP-chip, C2C12 cells were transfected with Flag-DPF3a and ChIP was performed using anti-Flag antibody.

Method	Cell type	Antibody for ChIP	Total peaks	Peaks with E-box
ChIP-chip	C1C12 undifferentiated	Flag	134	88
ChIP-seq	C1C12 undifferentiated	pDPF3a	777	709
ChIP-seq	C1C12 differentiated	pDPF3a	821	782
